# Supplementary figures and images for: AAV-mediated expression of proneural factors stimulates neurogenesis from adult Müller glia in vivo
Source: EMBO Mol Med. 2025 Mar 6;17(4):722–46. doi: 10.1038/s44321-025-00209-3 (PMC11982270; doi:10.1038/s44321-025-00209-3)

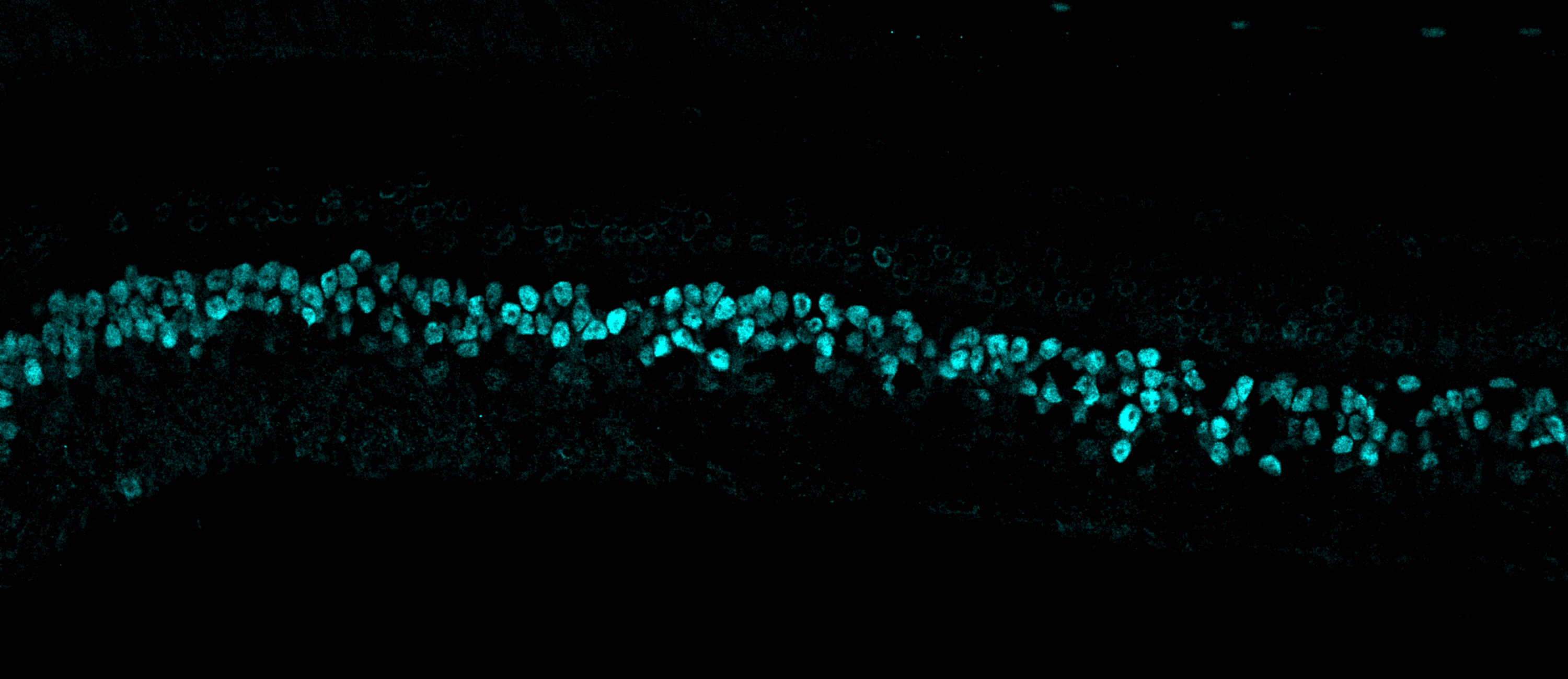

Supplement: Supplementary file 2 — Source data Fig. 1 [file 44321_2025_209_MOESM2_ESM.zip › Fig 1/CBh-FLEX[Ascl1]_Otx2.tif]

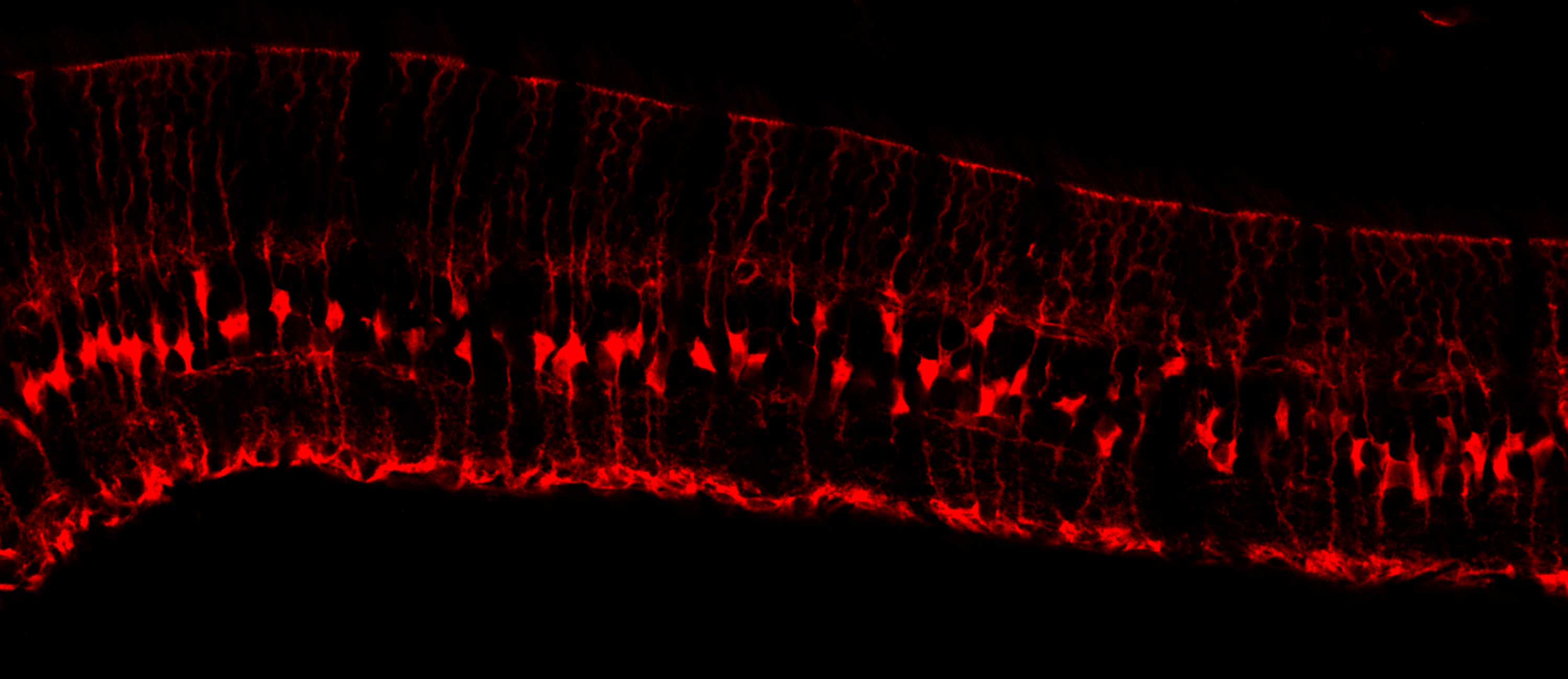

Supplement: Supplementary file 2 — Source data Fig. 1 [file 44321_2025_209_MOESM2_ESM.zip › Fig 1/CBh-FLEX[Ascl1]_TdT.tif]

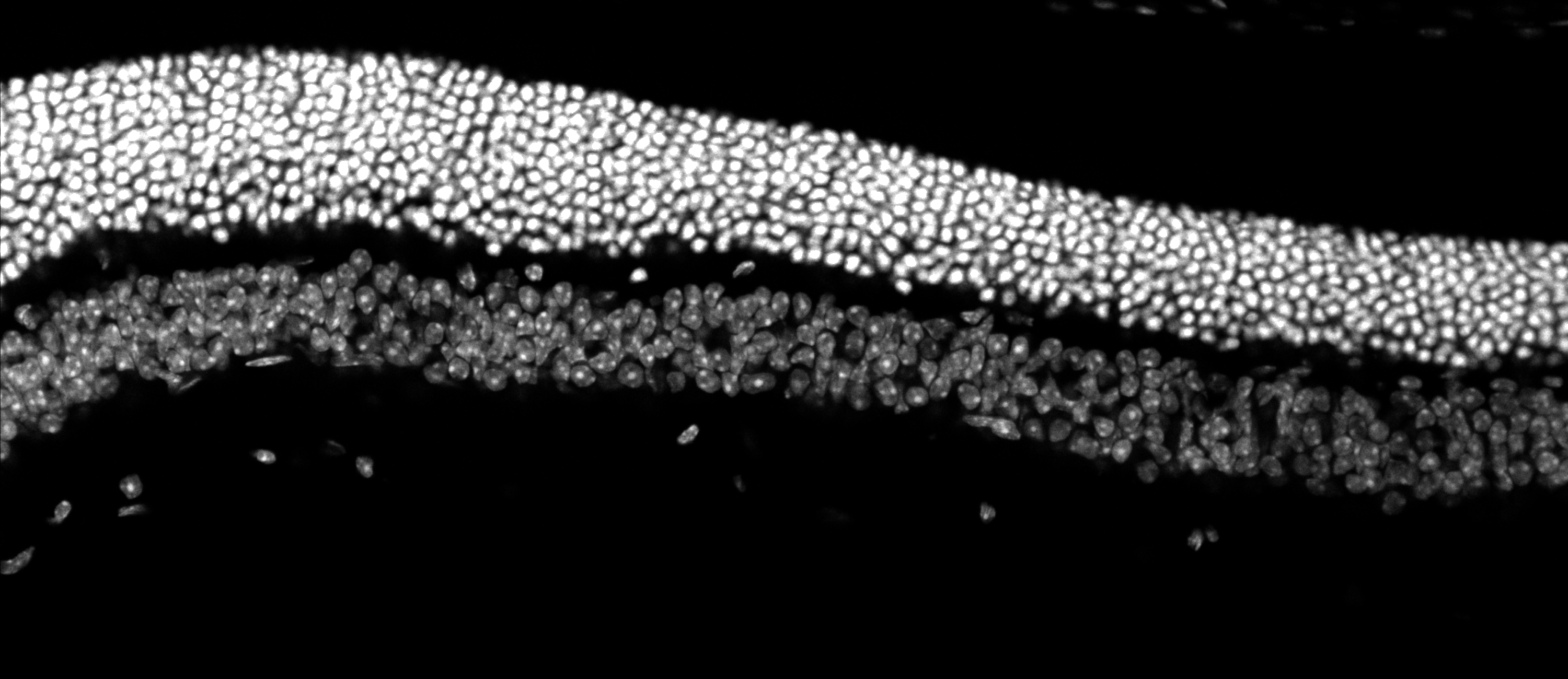

Supplement: Supplementary file 2 — Source data Fig. 1 [file 44321_2025_209_MOESM2_ESM.zip › Fig 1/CBh-FLEX[Ascl1]_DAPI.tif]

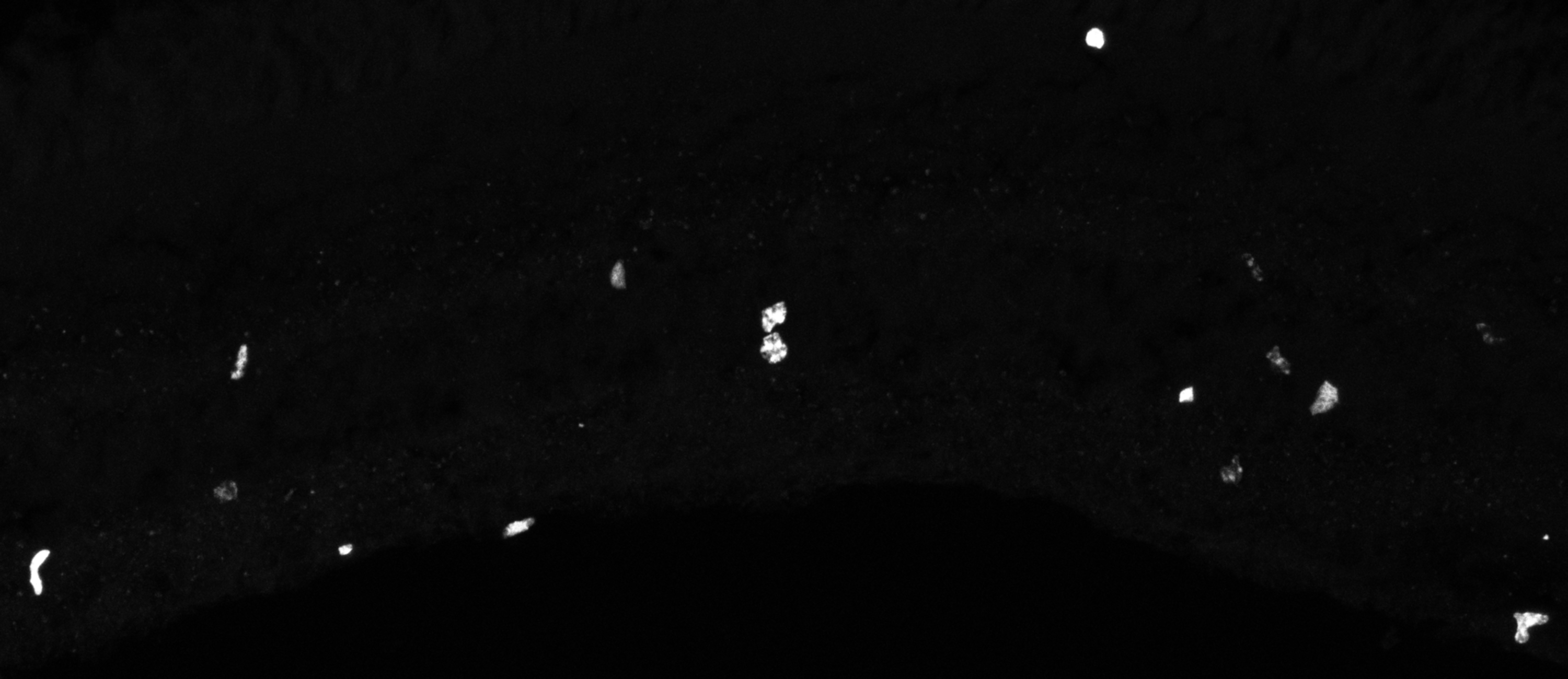

Supplement: Supplementary file 2 — Source data Fig. 1 [file 44321_2025_209_MOESM2_ESM.zip › Fig 1/CBh-FLEX[Ascl1](RFP,EdU).tif]

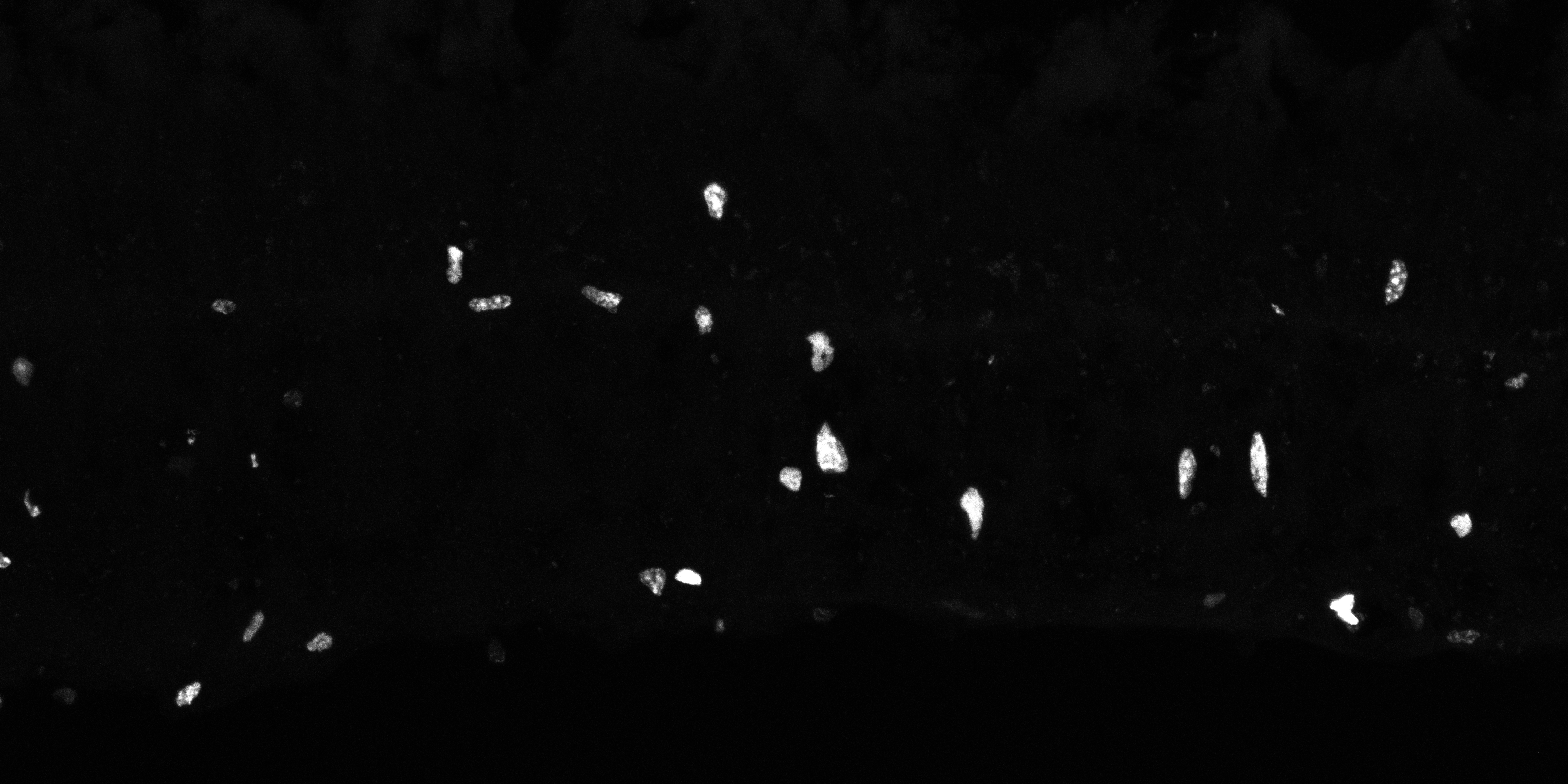

Supplement: Supplementary file 2 — Source data Fig. 1 [file 44321_2025_209_MOESM2_ESM.zip › Fig 1/Ef1a-FLEX[Ascl1](RFP,EdU).tif]

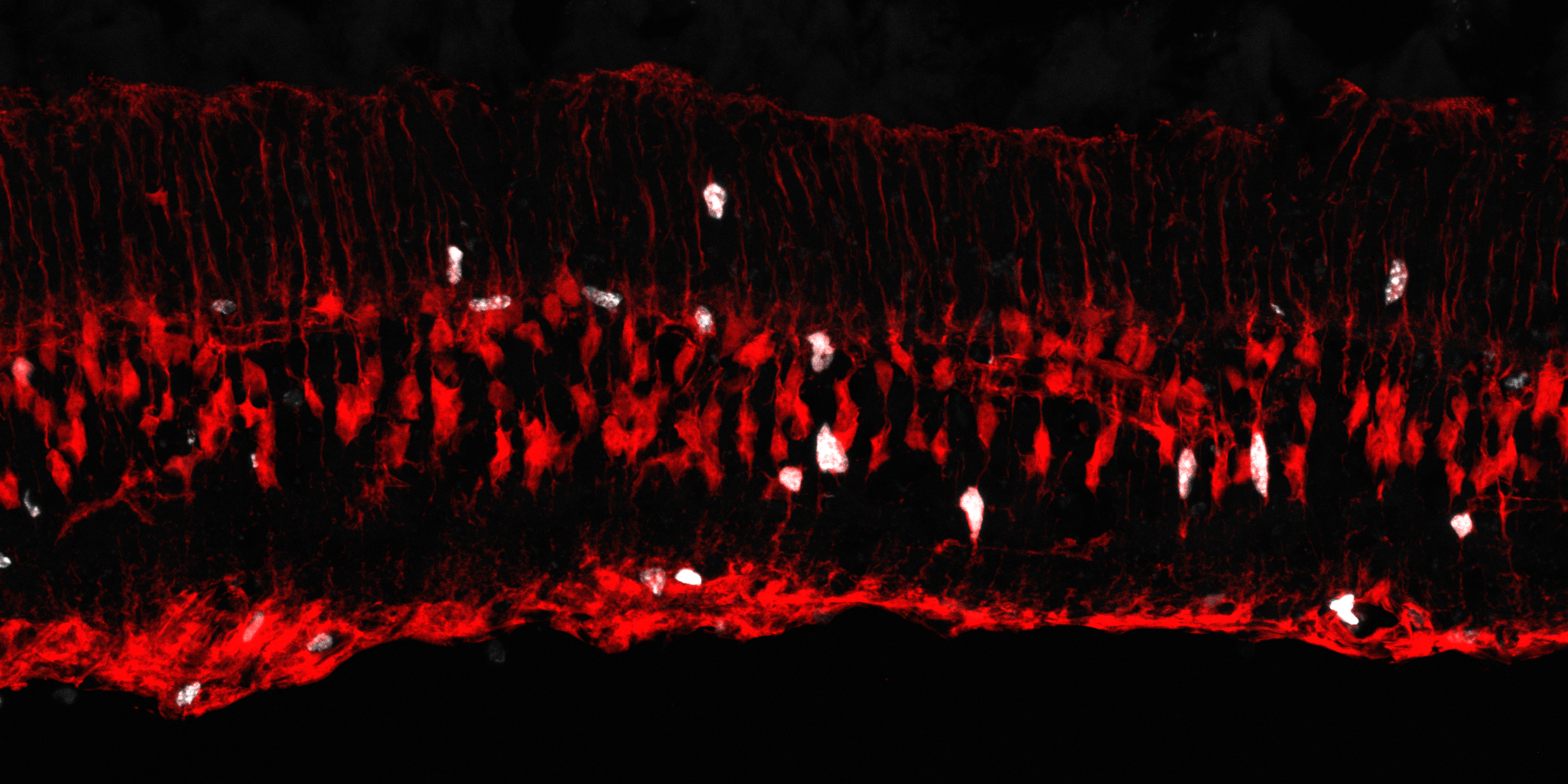

Supplement: Supplementary file 2 — Source data Fig. 1 [file 44321_2025_209_MOESM2_ESM.zip › Fig 1/Ef1a-FLEX[Ascl1](RFP,EdU)_RGB.tif]

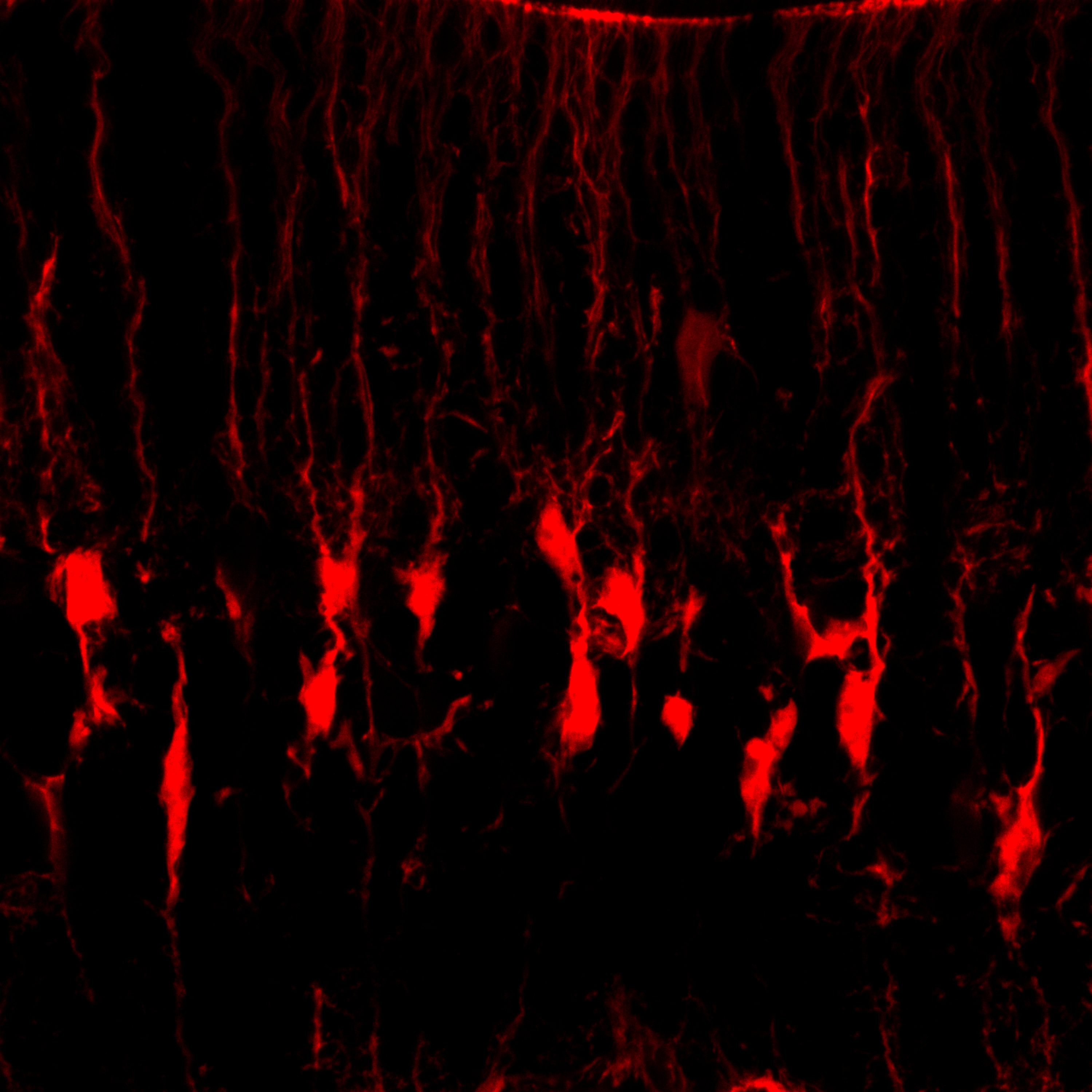

Supplement: Supplementary file 2 — Source data Fig. 1 [file 44321_2025_209_MOESM2_ESM.zip › Fig 1/F_CBh-FLEX[Ascl1]_TdT.tif]

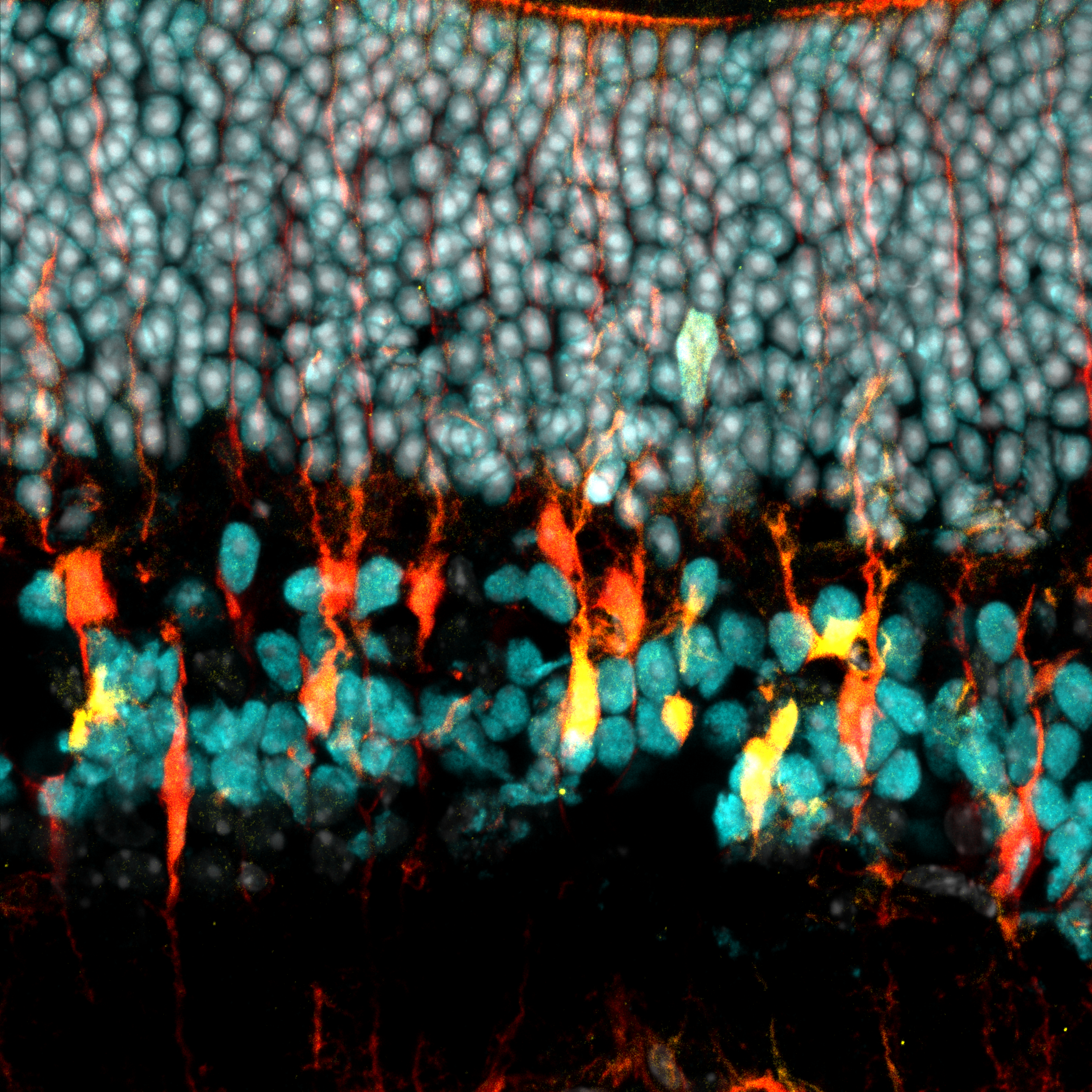

Supplement: Supplementary file 2 — Source data Fig. 1 [file 44321_2025_209_MOESM2_ESM.zip › Fig 1/F_CBh-FLEX[Ascl1]_RGB.tif]

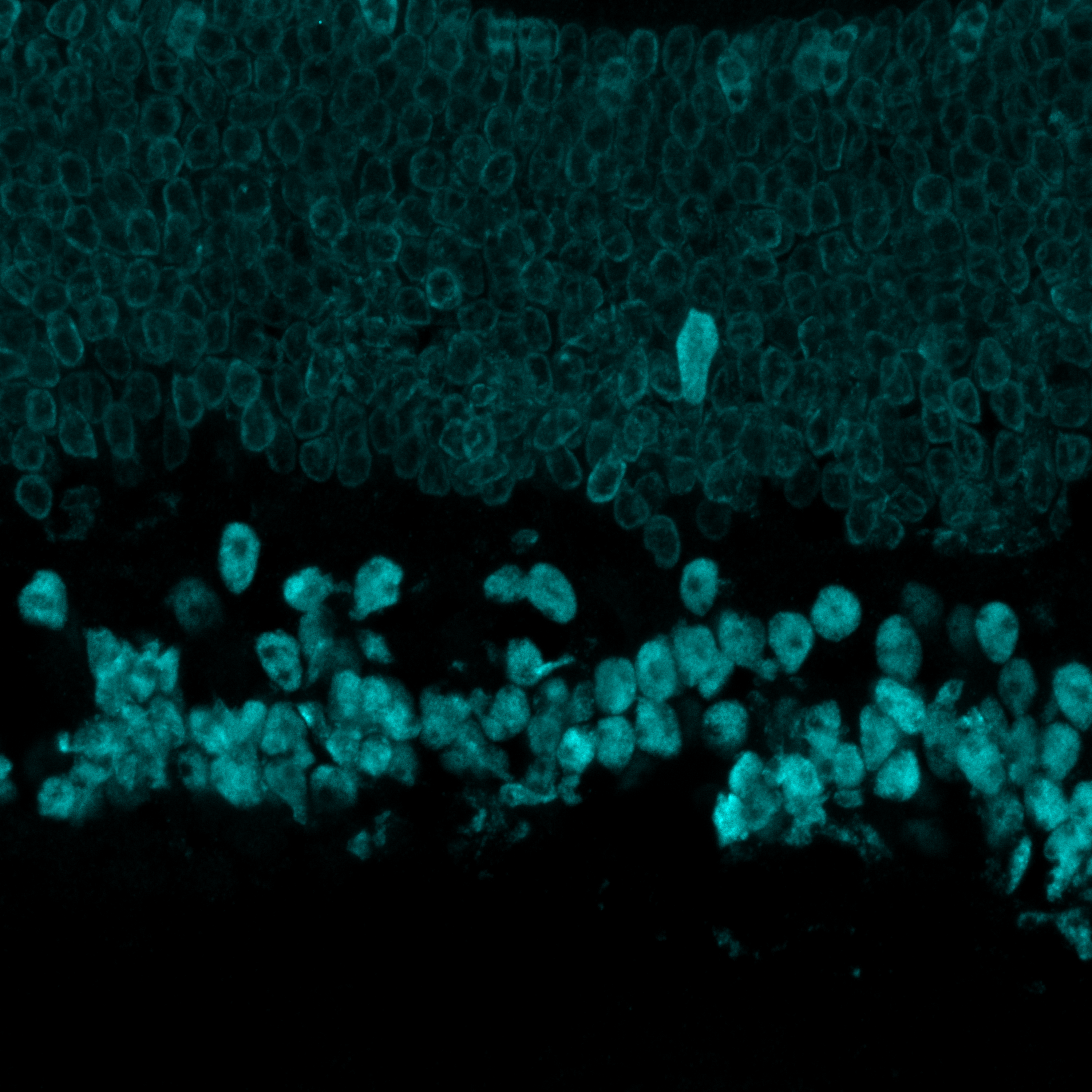

Supplement: Supplementary file 2 — Source data Fig. 1 [file 44321_2025_209_MOESM2_ESM.zip › Fig 1/F_CBh-FLEX[Ascl1]_Otx2.tif]

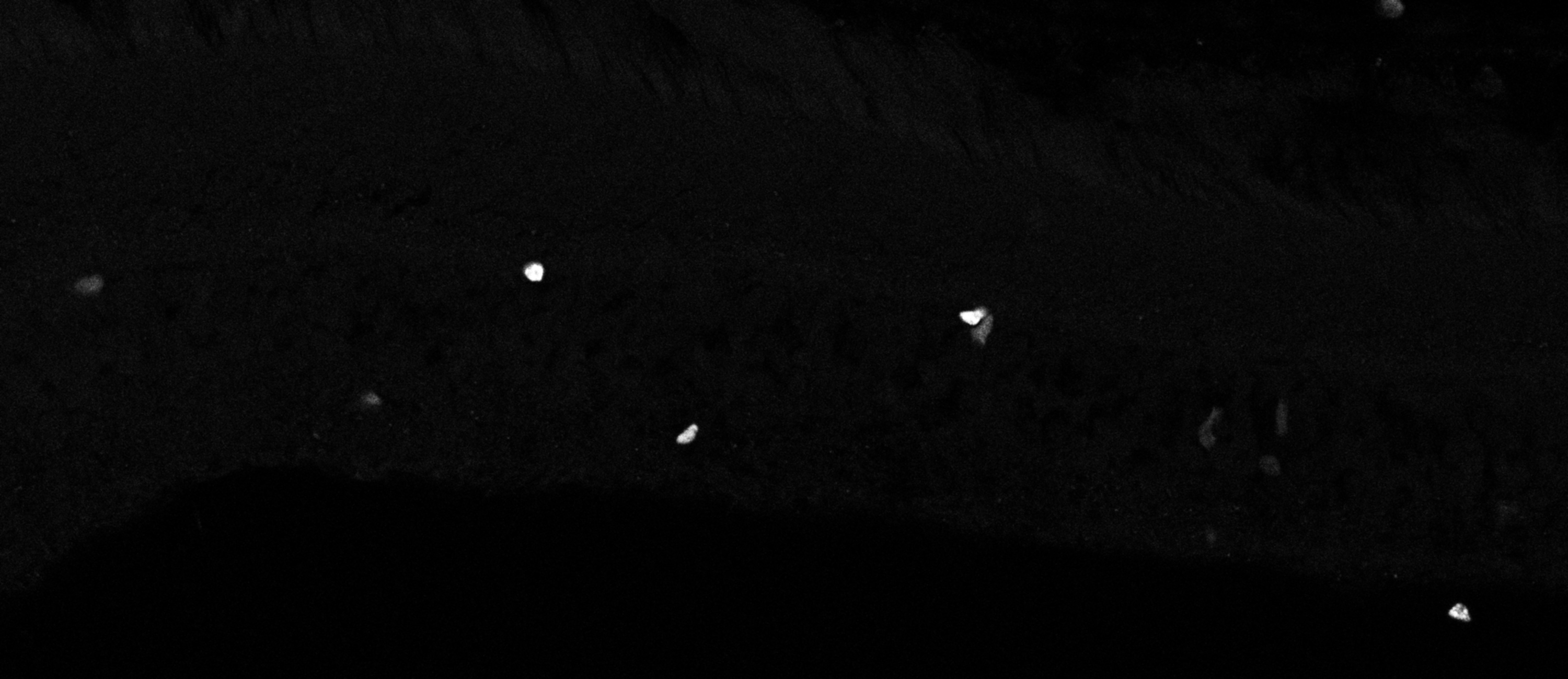

Supplement: Supplementary file 2 — Source data Fig. 1 [file 44321_2025_209_MOESM2_ESM.zip › Fig 1/CBh-FLEX[Ascl1].tif]

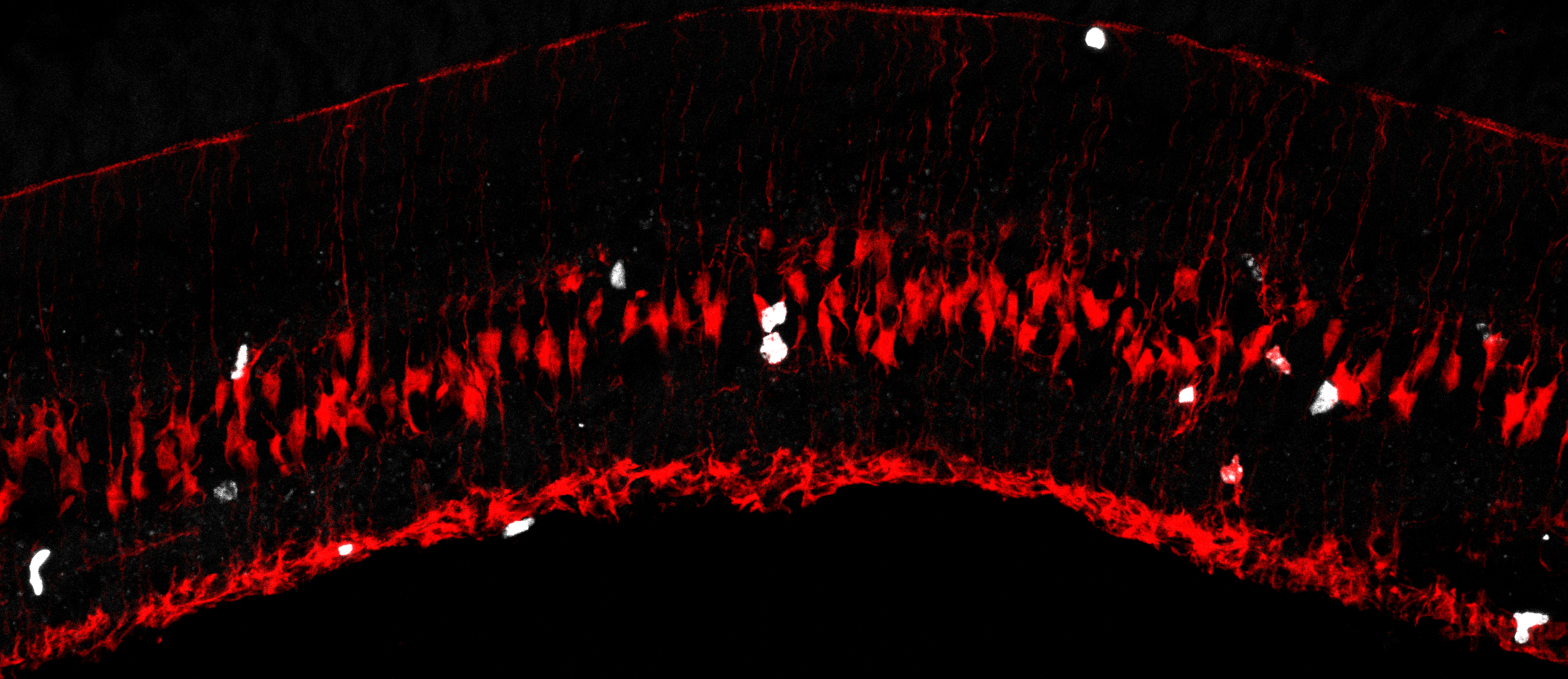

Supplement: Supplementary file 2 — Source data Fig. 1 [file 44321_2025_209_MOESM2_ESM.zip › Fig 1/CBh-FLEX[Ascl1](RFP,EdU)_RGB.tif]

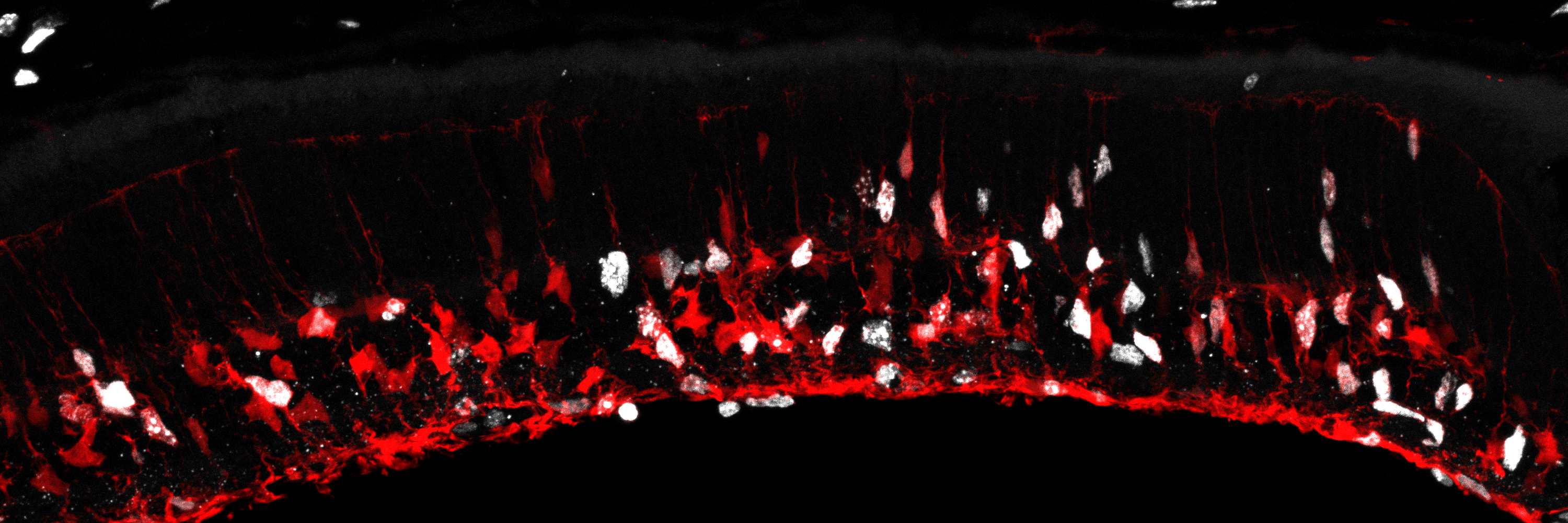

Supplement: Supplementary file 2 — Source data Fig. 1 [file 44321_2025_209_MOESM2_ESM.zip › Fig 1/htCBh-FLEX[Ascl1](RFP,EdU)_RGB.tif]

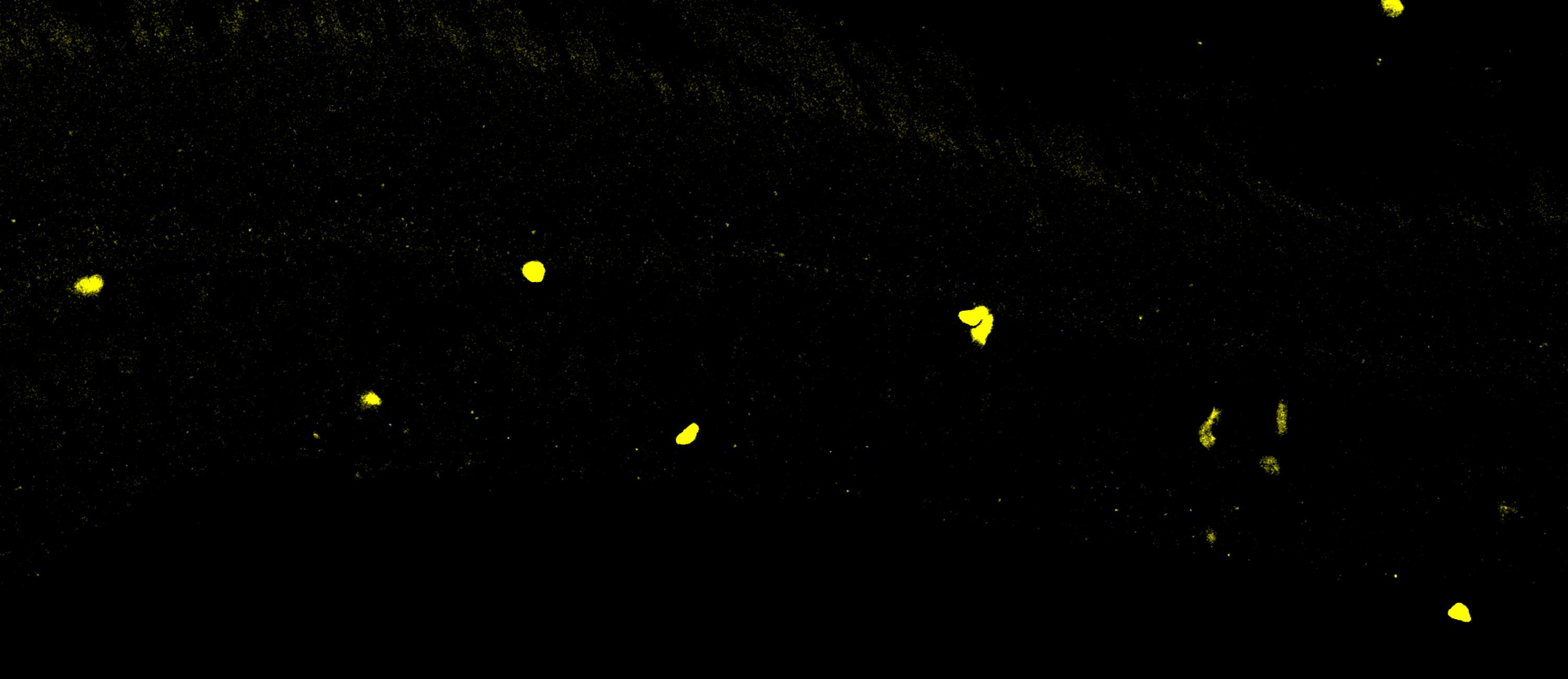

Supplement: Supplementary file 2 — Source data Fig. 1 [file 44321_2025_209_MOESM2_ESM.zip › Fig 1/CBh-FLEX[Ascl1]_EdU.tif]

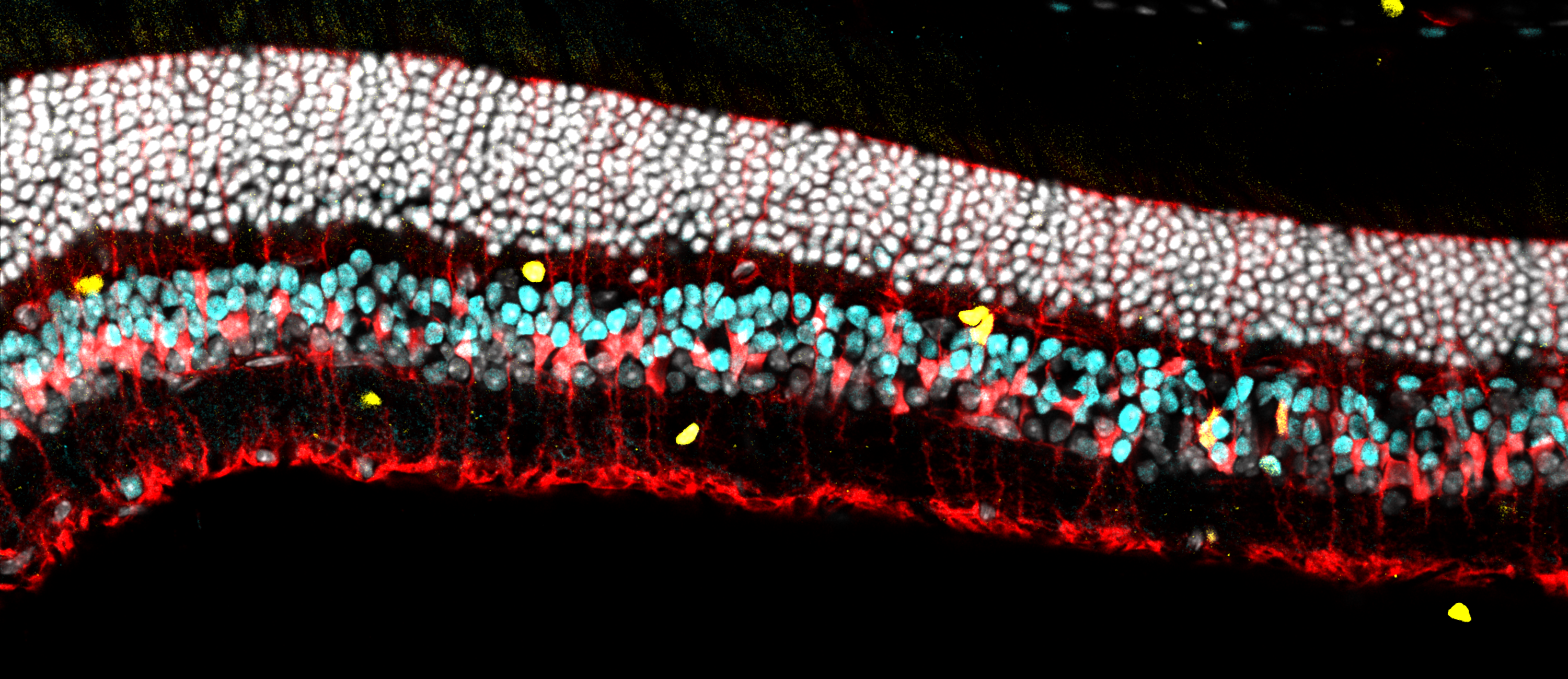

Supplement: Supplementary file 2 — Source data Fig. 1 [file 44321_2025_209_MOESM2_ESM.zip › Fig 1/CBh-FLEX[Ascl1]_RGB.tif]

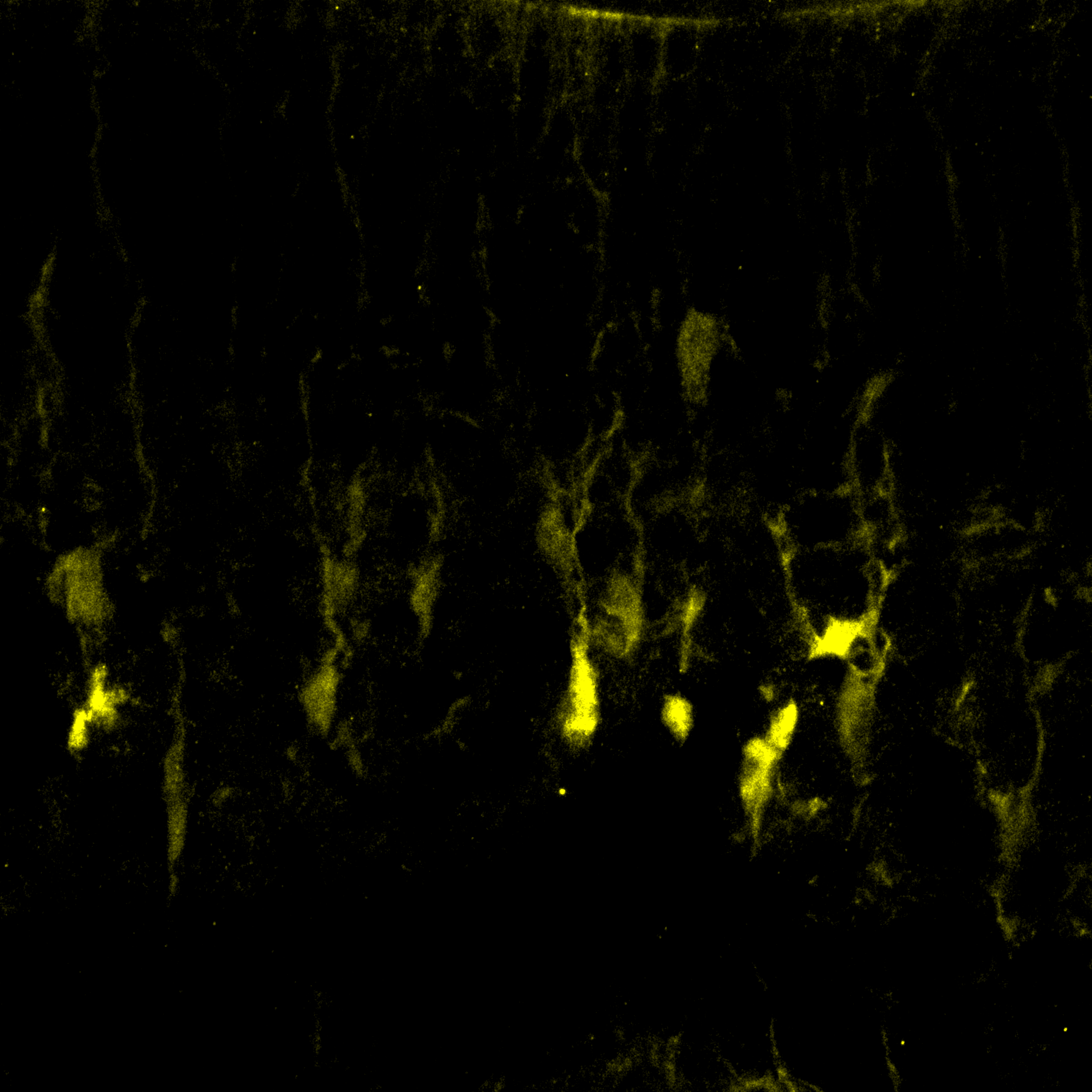

Supplement: Supplementary file 2 — Source data Fig. 1 [file 44321_2025_209_MOESM2_ESM.zip › Fig 1/CBh-FLEX[Ascl1]_Ascl1-GFP.tif]

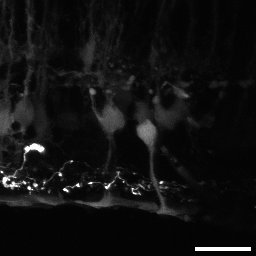

Supplement: Supplementary file 3 — Source data Fig. 2 [file 44321_2025_209_MOESM3_ESM.zip › Fig 2/MAX_20240725Bc5.ids (RGB).tif]

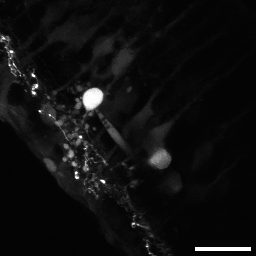

Supplement: Supplementary file 3 — Source data Fig. 2 [file 44321_2025_209_MOESM3_ESM.zip › Fig 2/MAX_20240725Bc8.ids (RGB).tif]

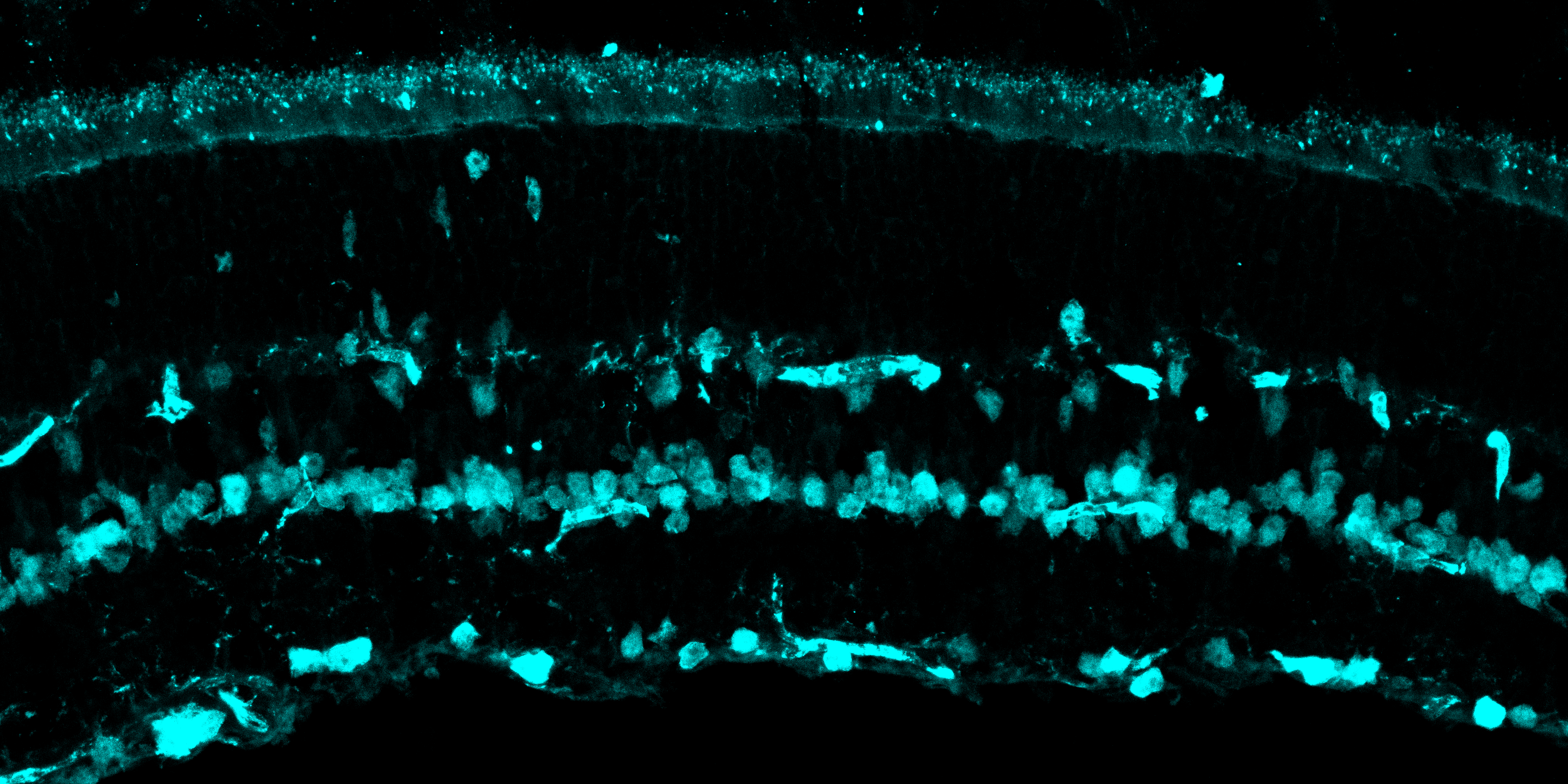

Supplement: Supplementary file 4 — Source data Fig. 3 [file 44321_2025_209_MOESM4_ESM.zip › Fig 3/CBh-FLEX[Ascl1-Atoh1]_HuCD.tif]

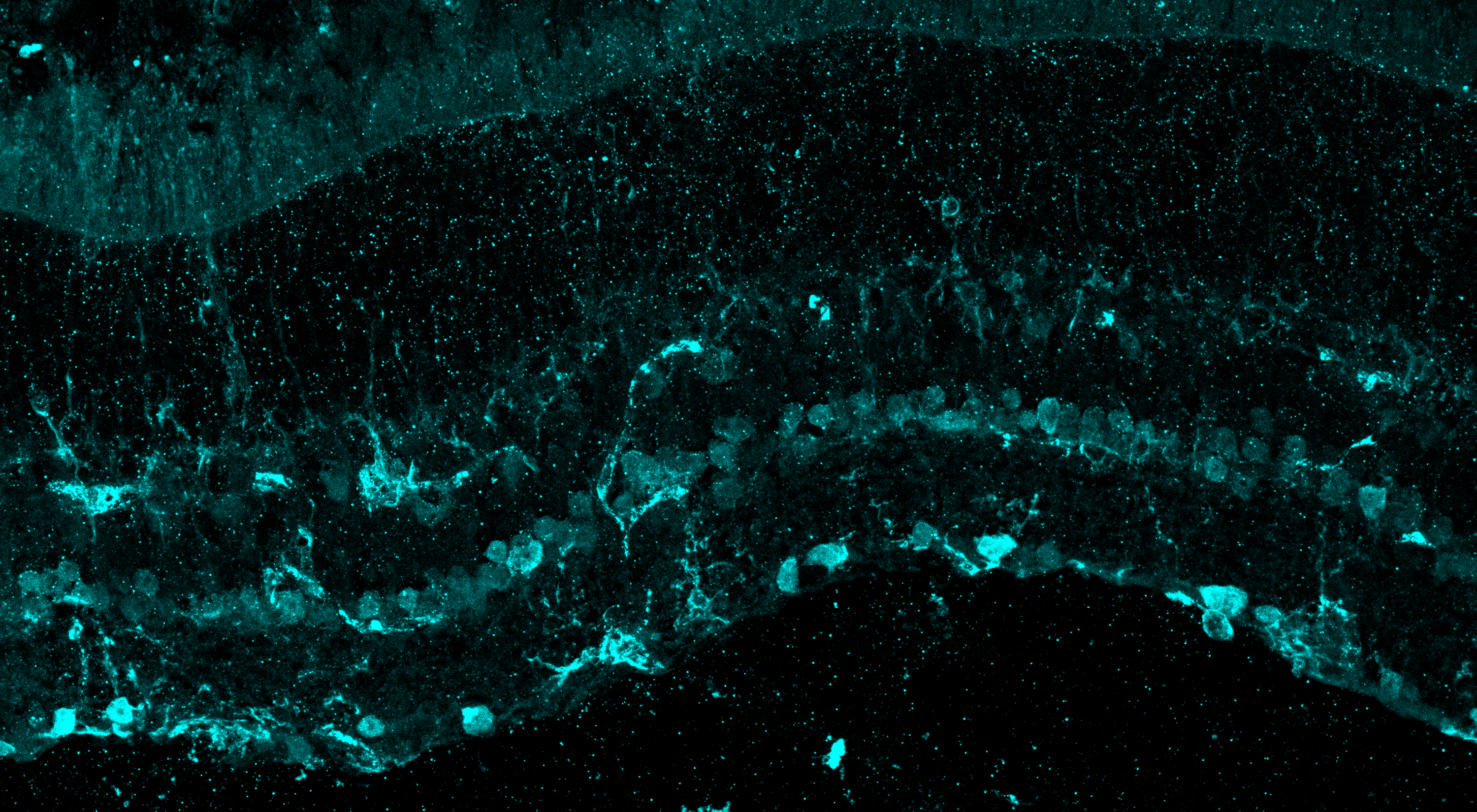

Supplement: Supplementary file 4 — Source data Fig. 3 [file 44321_2025_209_MOESM4_ESM.zip › Fig 3/CBh-FLEX[Ascl1-Atoh7]_HuCD.tif]

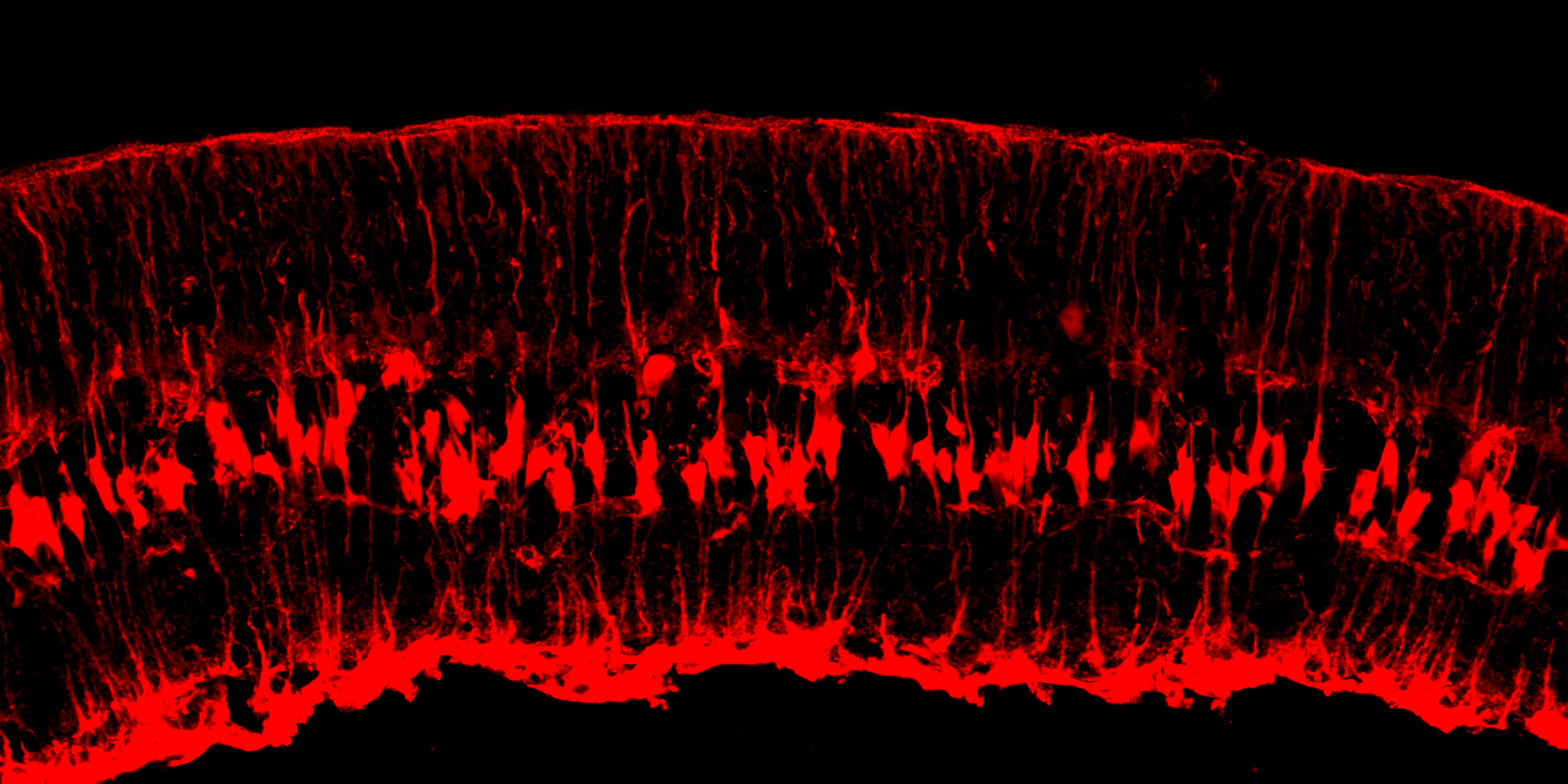

Supplement: Supplementary file 4 — Source data Fig. 3 [file 44321_2025_209_MOESM4_ESM.zip › Fig 3/CBh-FLEX[Ascl1-Atoh1]_TdT.tif]

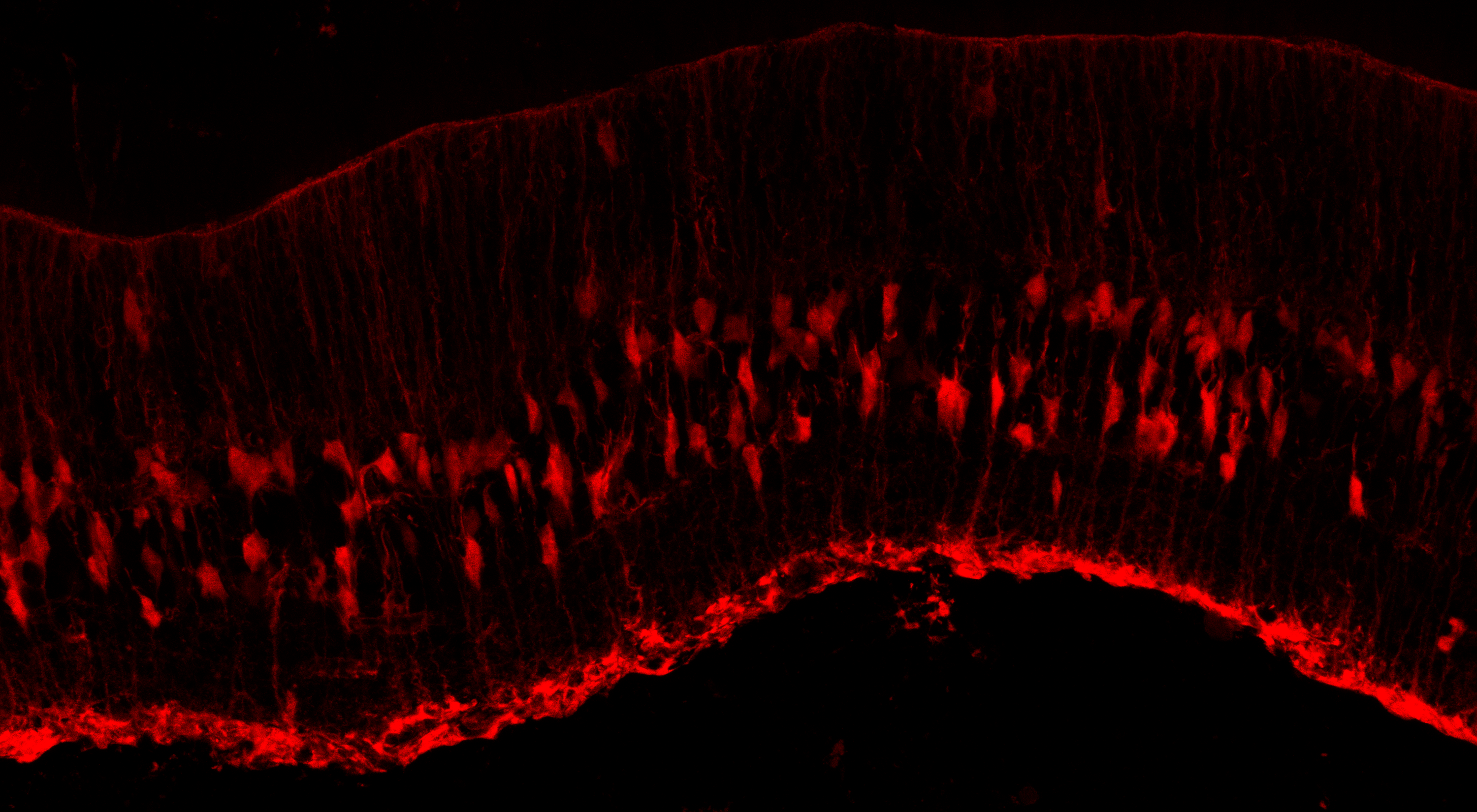

Supplement: Supplementary file 4 — Source data Fig. 3 [file 44321_2025_209_MOESM4_ESM.zip › Fig 3/CBh-FLEX[Ascl1-Atoh7]_TdT.tif]

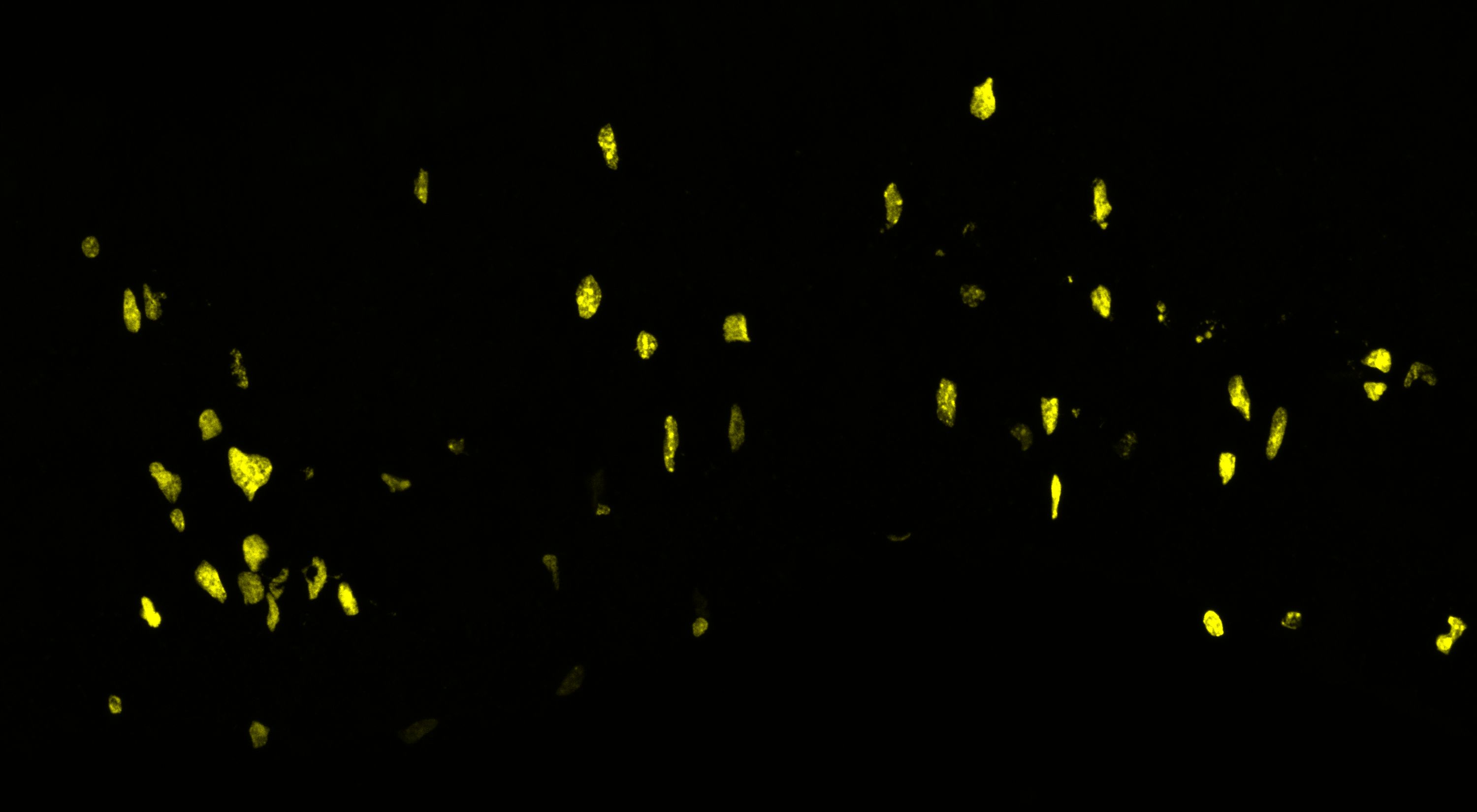

Supplement: Supplementary file 4 — Source data Fig. 3 [file 44321_2025_209_MOESM4_ESM.zip › Fig 3/CBh-FLEX[Ascl1-Atoh7]_EdU.tif]

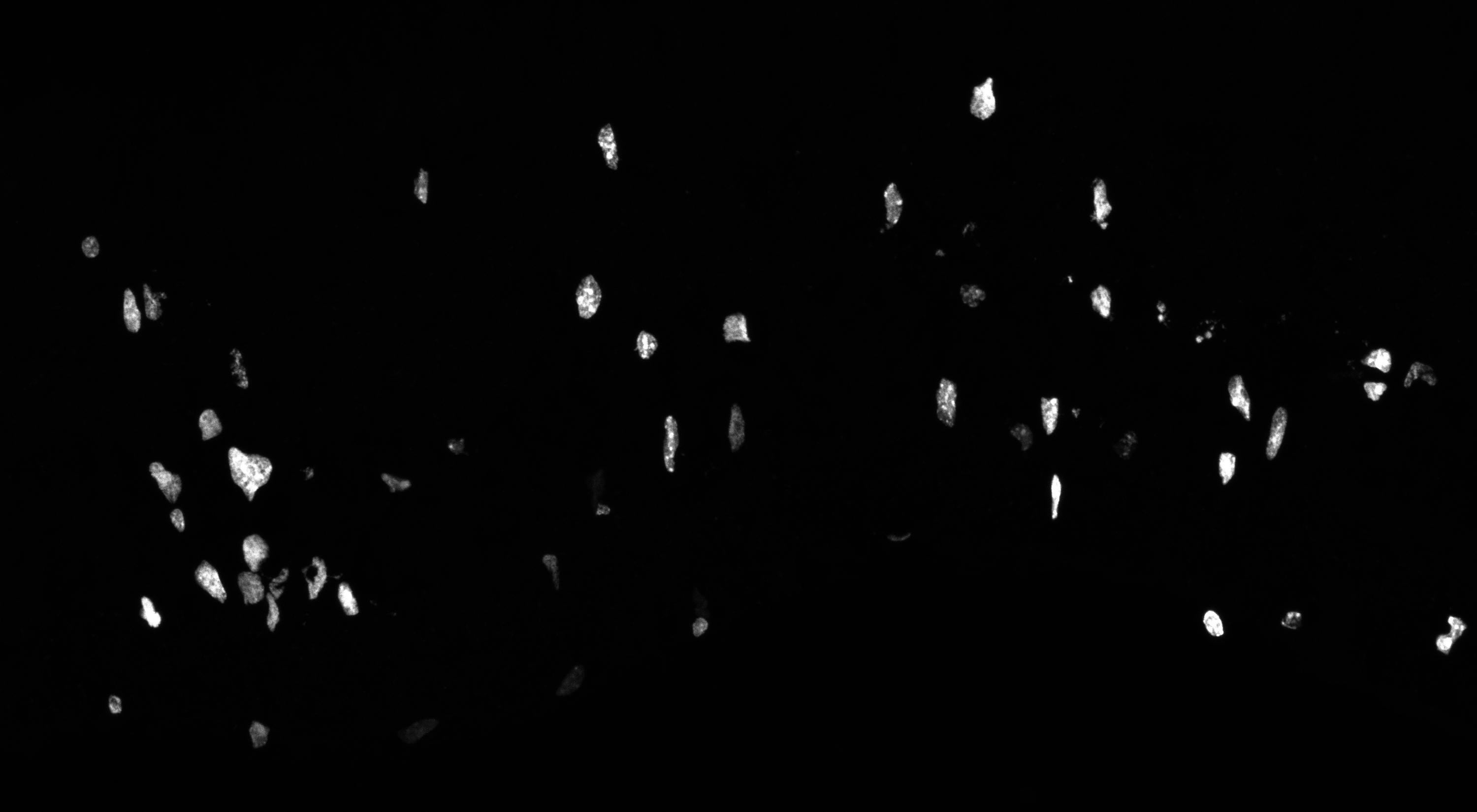

Supplement: Supplementary file 4 — Source data Fig. 3 [file 44321_2025_209_MOESM4_ESM.zip › Fig 3/CBh-FLEX[Ascl1-Atoh7]_RGB.tif]

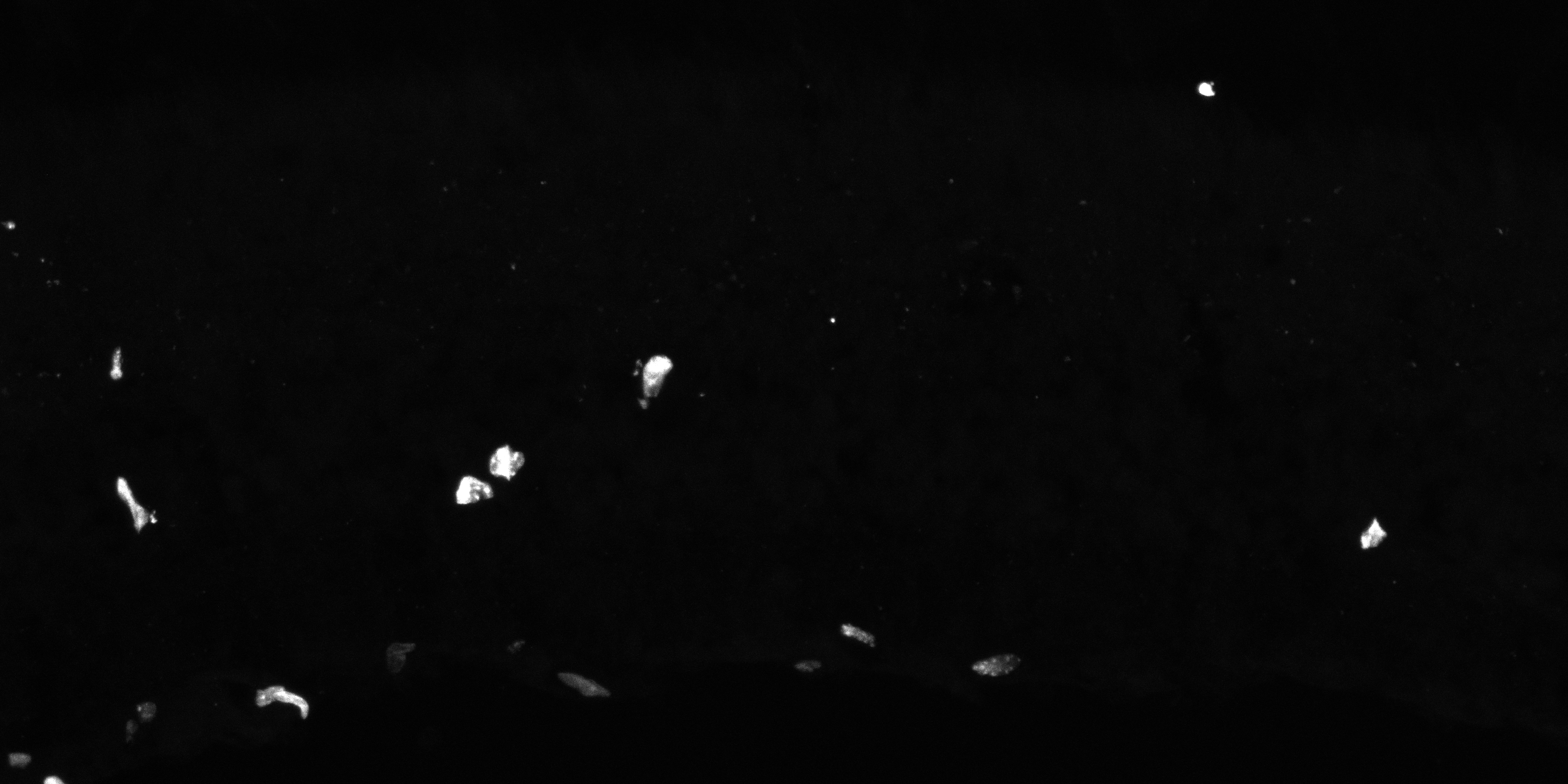

Supplement: Supplementary file 4 — Source data Fig. 3 [file 44321_2025_209_MOESM4_ESM.zip › Fig 3/CBh-FLEX[Ascl1-Atoh1].tif]

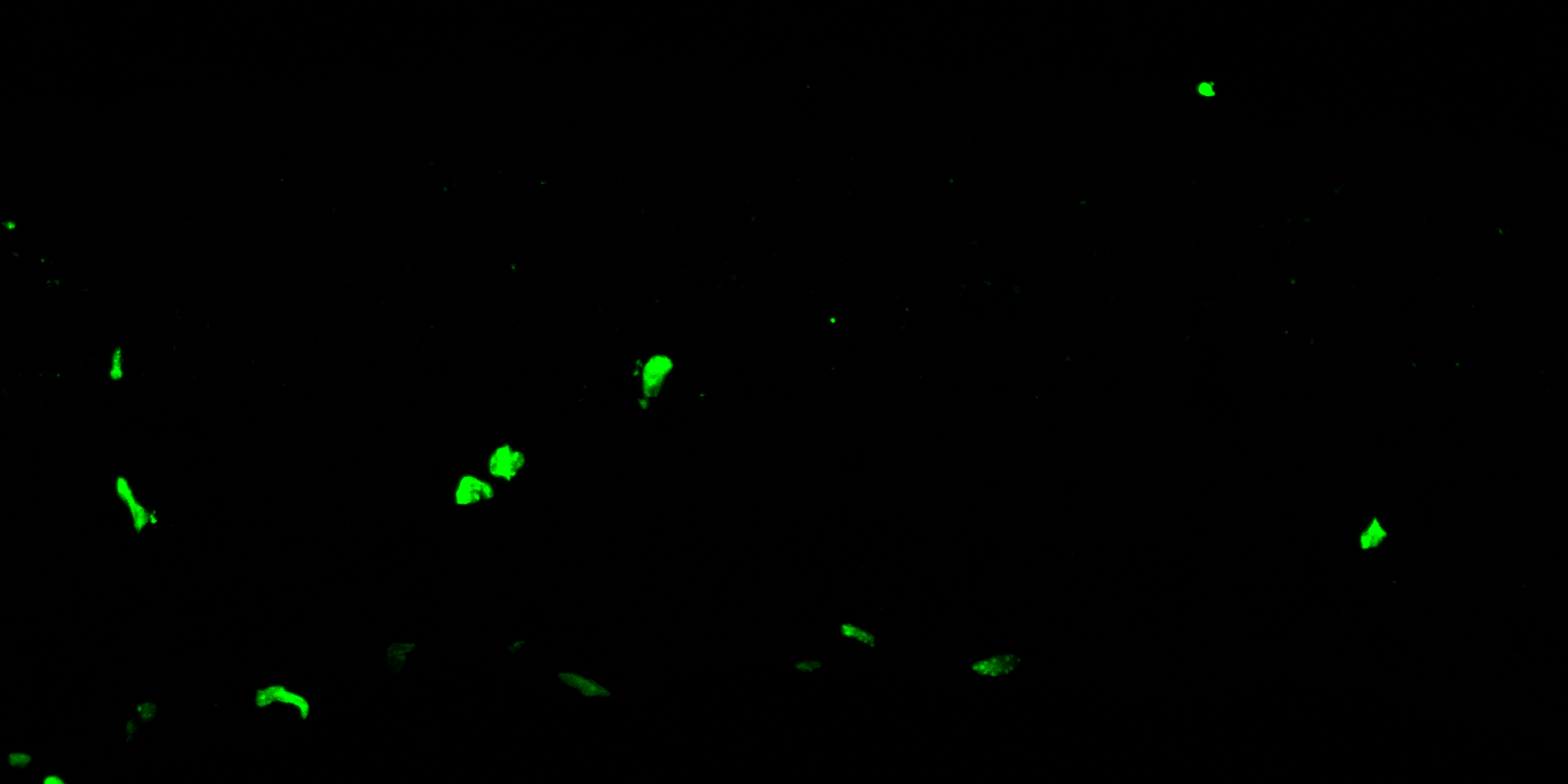

Supplement: Supplementary file 4 — Source data Fig. 3 [file 44321_2025_209_MOESM4_ESM.zip › Fig 3/CBh-FLEX[Ascl1-Atoh1]_EdU.tif]

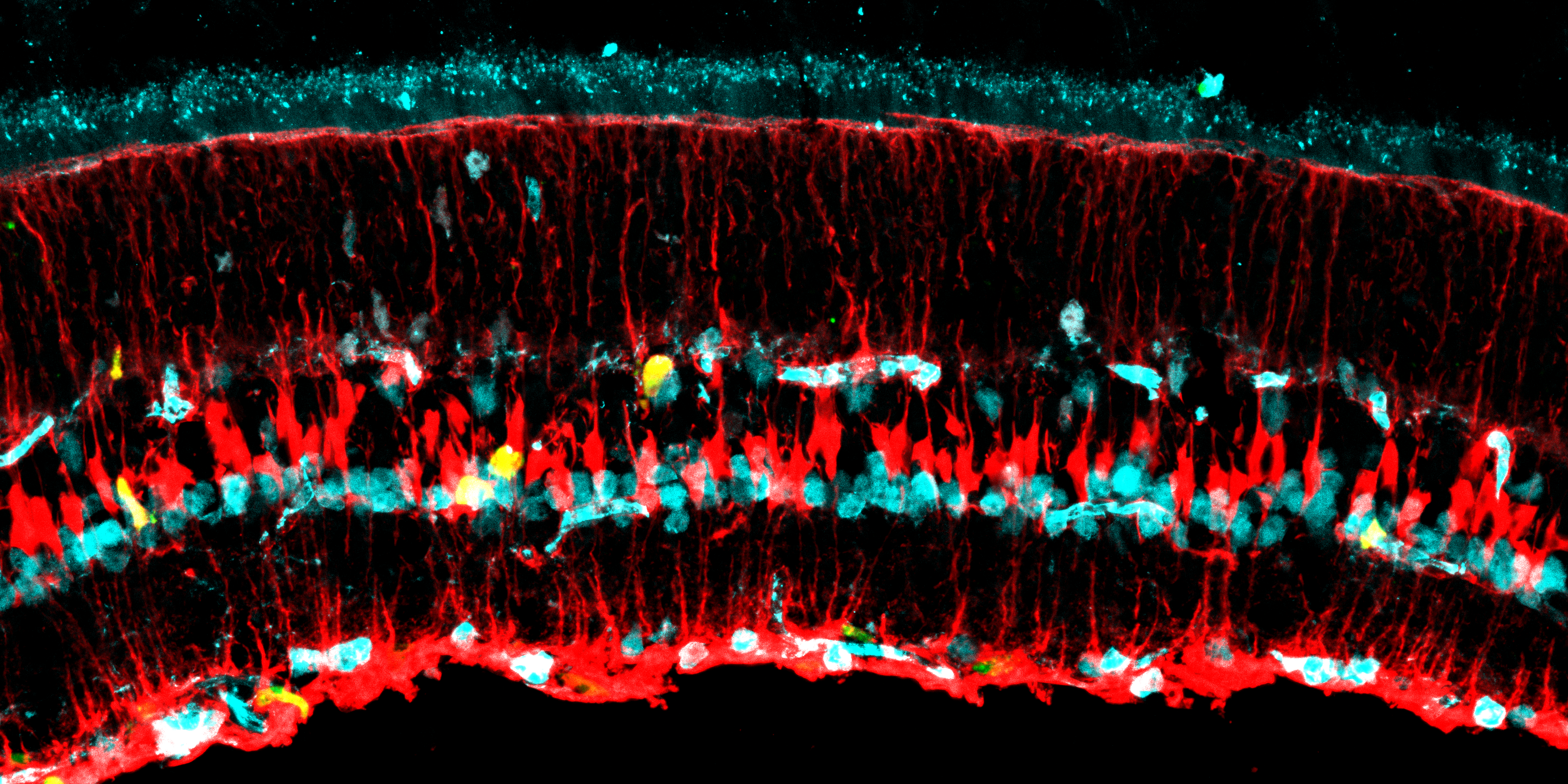

Supplement: Supplementary file 4 — Source data Fig. 3 [file 44321_2025_209_MOESM4_ESM.zip › Fig 3/CBh-FLEX[Ascl1-Atoh1]_RGB.tif]

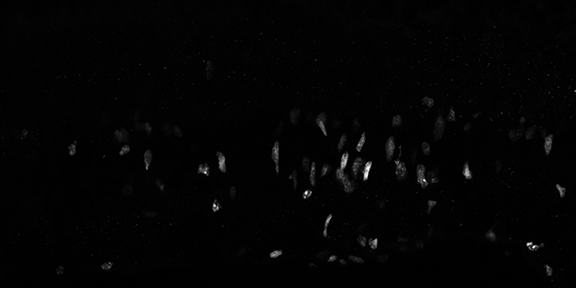

Supplement: Supplementary file 5 — Source data Fig. 4 [file 44321_2025_209_MOESM5_ESM.zip › Fig 4/CBh-FLEX[Ascl1-Atoh7].tif]

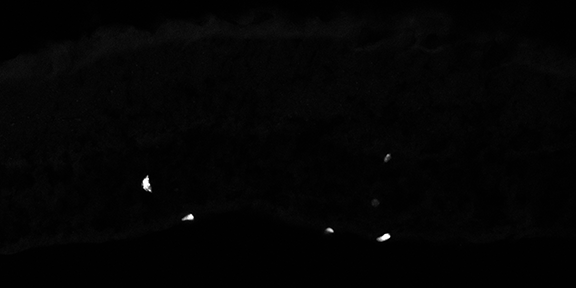

Supplement: Supplementary file 5 — Source data Fig. 4 [file 44321_2025_209_MOESM5_ESM.zip › Fig 4/CBh-FLEX[Ascl1].tif]
