# Supplementary material for: AAV-mediated expression of proneural factors stimulates neurogenesis from adult Müller glia in vivo
Source: EMBO Mol Med. 2025 Mar 6;17(4):722–46. doi: 10.1038/s44321-025-00209-3 (PMC11982270; doi:10.1038/s44321-025-00209-3)
Supplement: Supplementary file 6 — Expanded View Figures [file 44321_2025_209_MOESM6_ESM.pdf]

Expanded View Figures

Figure EV1. Assessment of transgenic lineage tracer mouse line and AAV kinetics in vivo.

(**A**) Schematic of tamoxifen-inducible transgenic mouse line for MG-specific expression, (**B**) bar plot of min to max quantification of recombination efficiency counted as percentage ratio of $RFP^{+}Sox2^{+}$ cells over all $Sox2^{+}$ where each dot is a biological replicate (error bar: mean plus standard deviation), (**C–C**″) fluorescence images of Rlbp1-CreERt2 x LSL-TdT retina cross-sections after tamoxifen showing DAPI-stained nuclei in white, TdT in red and HuC/D in cyan, (**D–D**″) fluorescence images of Rlbp1-CreERt2 x LSL-TdT retina cross-sections after tamoxifen showing DAPI-stained nuclei in white, TdT in red, Otx2 in blue and EdU in yellow, (**E**) fluorescence image of an untreated retina flatmount with TdT in red and vasculature in white, (E′) representative retina cross-section of untreated retina with TdT in red and Sox2 in yellow (**F**) schematic of experimental design, (**G**) fluorescence images of retina flatmounts for each timepoint with TdT in red and vasculature in white, (**G′**) representative retina cross-sections from timepoints in (**G**) with DAPI-stained nuclei in white, TdT in red and Sox2 in yellow; scalebars for (**C–C″**, **D–D″**, **G′**, **E′**): 50 µm, for (**E**, **G**): 500 µm, ONL outer nuclear layer, INL inner nuclear layer, GCL ganglion cell layer.

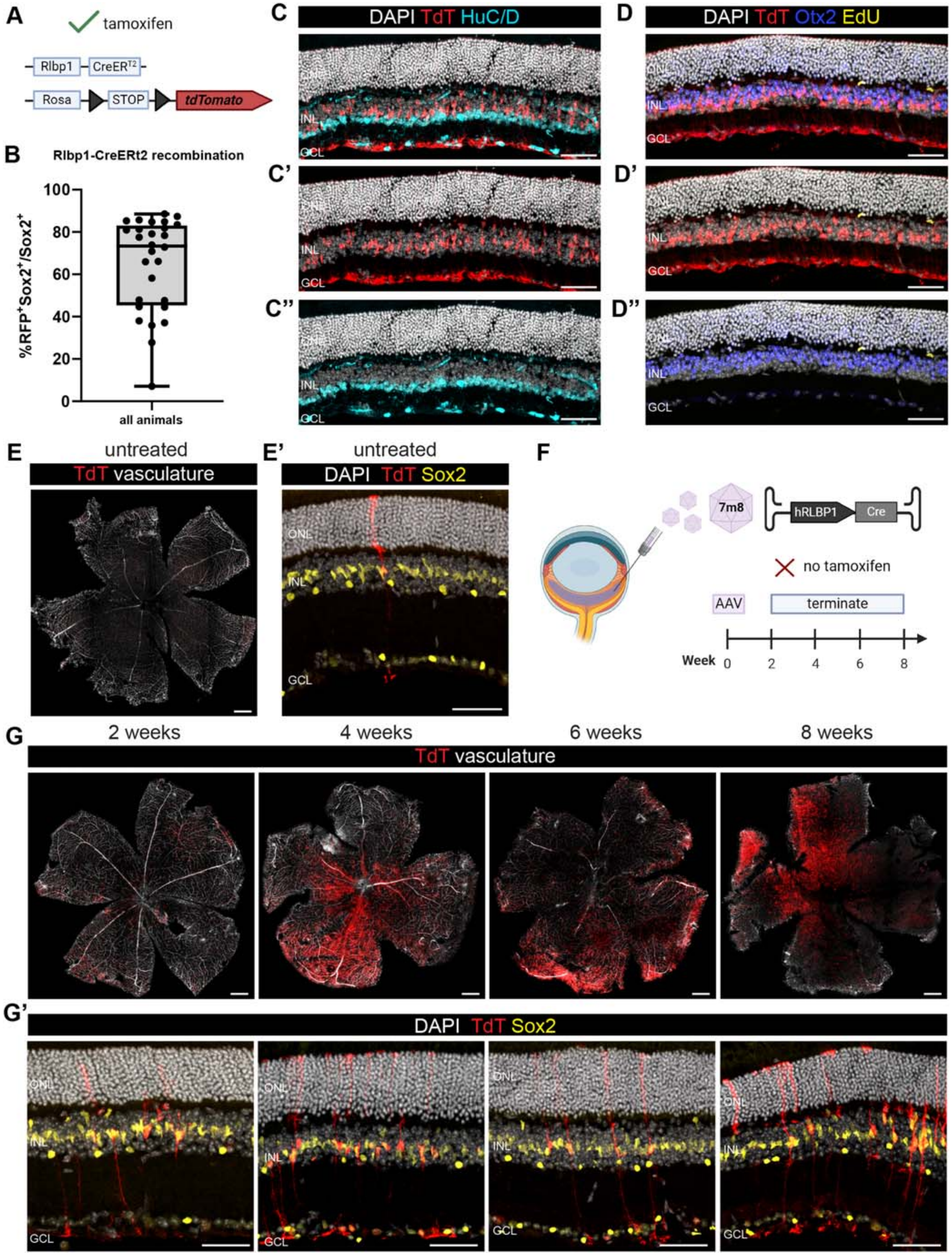
A
tamoxifen
Rlbp1
CreER^T2
Rosa
STOP
tdTomato
B
Rlbp1-CreERt2 recombination
%RFP+Sox2+/Sox2+
100
80
60
40
20
0
all animals
C
DAPI TdT HuC/D
C'
C''
D
DAPI TdT Otx2 EdU
D'
D''
ONL
INL
GCL
E
untreated
TdT vasculature
E'
untreated
DAPI TdT Sox2
F
7m8
hRLBP1
Cre
no tamoxifen
AAV
terminate
Week
0
2
4
6
8
G
2 weeks
4 weeks
6 weeks
8 weeks
TdT vasculature
G'
DAPI TdT Sox2

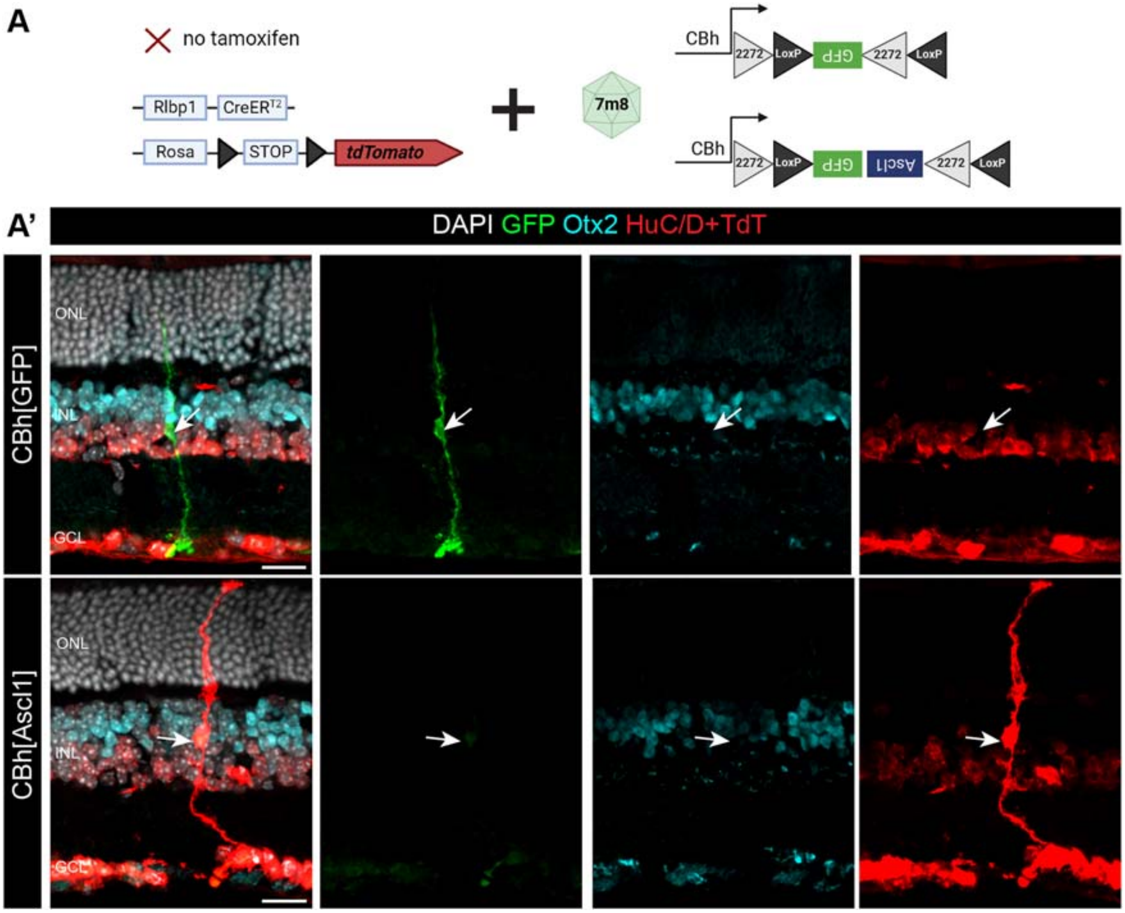

Figure EV2. Control experiments to assess Cre-dependent vector expression.

(**A**) Schematic of transgenic lineage tracer without tamoxifen administration and the FLEX vectors administered to assess traces of DNA recombination during AAV production, (**A'**) fluorescence images of stained tissue post CBh-FLEX[GFP] and CBh-FLEX[Ascl1-GFP] injection, with white arrows indicating cells where vector or lineage tracer recombination occurs without tamoxifen in MG cells that are not Otx2 or HuC/D positive.

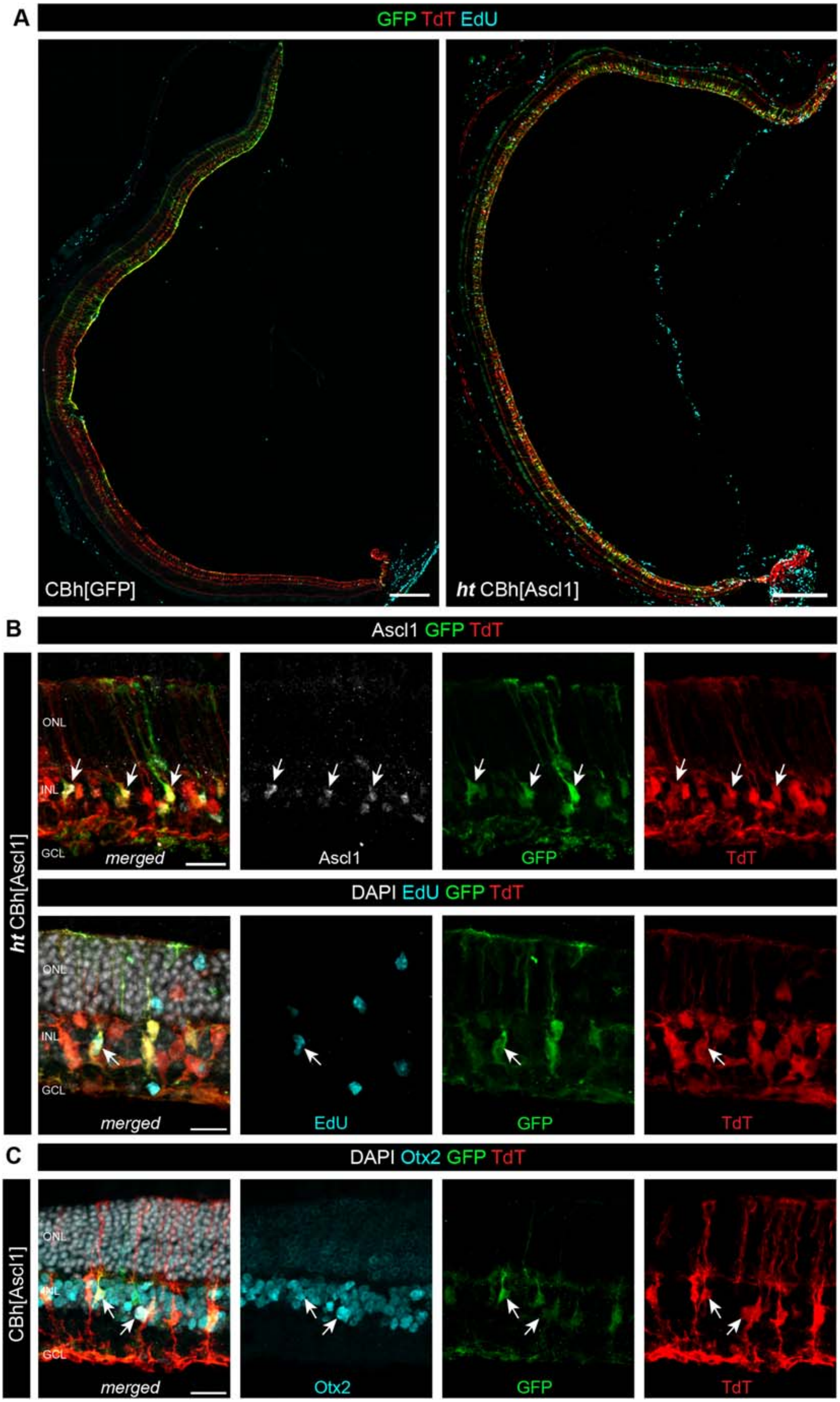
A
GFP TdT EdU
CBh[GFP]
ht CBh[Ascl1]
B
Ascl1 GFP TdT
ht CBh[Ascl1]
ONL
INL
GCL
merged
Ascl1
GFP
TdT
DAPI EdU GFP TdT
merged
EdU
GFP
TdT
C
DAPI Otx2 GFP TdT
CBh[Ascl1]
merged
Otx2
GFP
TdT

© The Author(s)

◀ **Figure EV3. AAV transduction and validation of protein markers.**

(**A**) Fluorescence images of Rlbp1-CreERt2 x LSL-TdT central retina cross-sections showing AAV-transduced cells in green, TdT in red and EdU proliferating cells in cyan for control and reprogramming vector conditions, (**B**) fluorescence images after reprogramming with ht AAV/CBh-FLEX[Ascl1], top row: transduced cells (white arrowhead) co-labeled with Ascl1 in white, GFP in green and TdT in red; bottom panel: transduced cells (white arrowhead) co-labeled with EdU in cyan, GFP in green and TdT in red, (**C**) fluorescence images after reprogramming with AAV/CBh-FLEX[Ascl1] showing transduced cells (white arrowhead) co-labeled with Otx2 in cyan, GFP in green and TdT in red; scale bar for (**A**): 200 µm, (**B**, **C**): 20 µm.

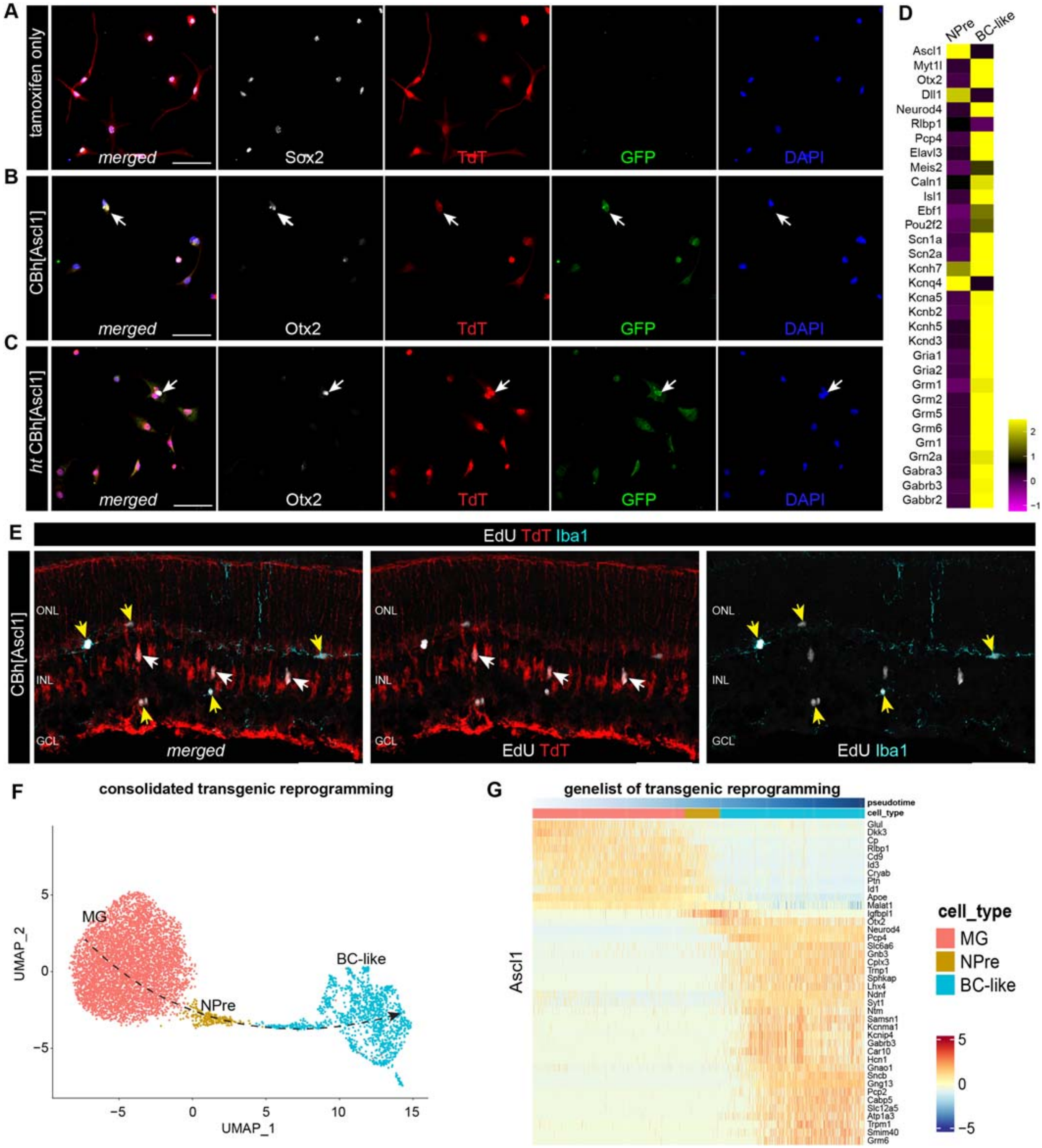
A
tamoxifen only
merged
Sox2
TdT
GFP
DAPI
B
CBh[Ascl1]
merged
Otx2
TdT
GFP
DAPI
C
ht CBh[Ascl1]
merged
Otx2
TdT
GFP
DAPI
D
NPre
BC-like
Ascl1
Myt1l
Otx2
Dll1
Neurod4
Rlbp1
Pcp4
Elavl3
Meis2
Caln1
Isl1
Ebf1
Pou2f2
Scn1a
Scn2a
Kcnh7
Kcnq4
Kcna5
Kcnb2
Kcnh5
Kcnd3
Gria1
Gria2
Grm1
Grm2
Grm5
Grm6
Grn1
Grn2a
Gabra3
Gabrb3
Gabbr2
E
EdU TdT Iba1
CBh[Ascl1]
ONL
INL
GCL
merged
EdU TdT
EdU Iba1
F
consolidated transgenic reprogramming
MG
NPre
BC-like
UMAP_1
UMAP_2
G
genelist of transgenic reprogramming
pseudotime
cell_type
Ascl1
cell_type
MG
NPre
BC-like

Figure EV4. Transcriptional analysis of AAV-mediated reprogramming.

(**A–C**) Fluorescence images of sorted lineage-traced cells on coverslips 24 h post FACS split by condition (white arrows indicate reprogrammed cells) showing merged and single channels of DAPI in blue, GFP vector reporter in green, TdT lineage tracer in red and glial marker Sox2 or bipolar marker Otx2 in white, (**D**) heatmap of average gene expression for selected neuronal genes in clusters NPre and MG-derived neurons following AAV-mediated reprogramming, (**E**) fluorescence images of retinal cross-section with proliferating lineage-traced MG (white arrows) and microglia (yellow arrows) with TdT in red, Iba1 in cyan and EdU in white, (**F**) UMAP of consolidated scRNA-seq data of Ascl1-mediated neurogenesis from transgenic animals (Glast-CreERt2 x LSL-tTA x tetO-Ascl1-GFP), (**G**) heatmap of top 40 differentially expressed genes across pseudotime trajectory from glial cell fate to neuronal cell fate based on scRNA-seq data from (**F**); scale bar: 50 µm, ONL outer nuclear layer, INL inner nuclear layer, GCL ganglion cell layer.

© The Author(s)

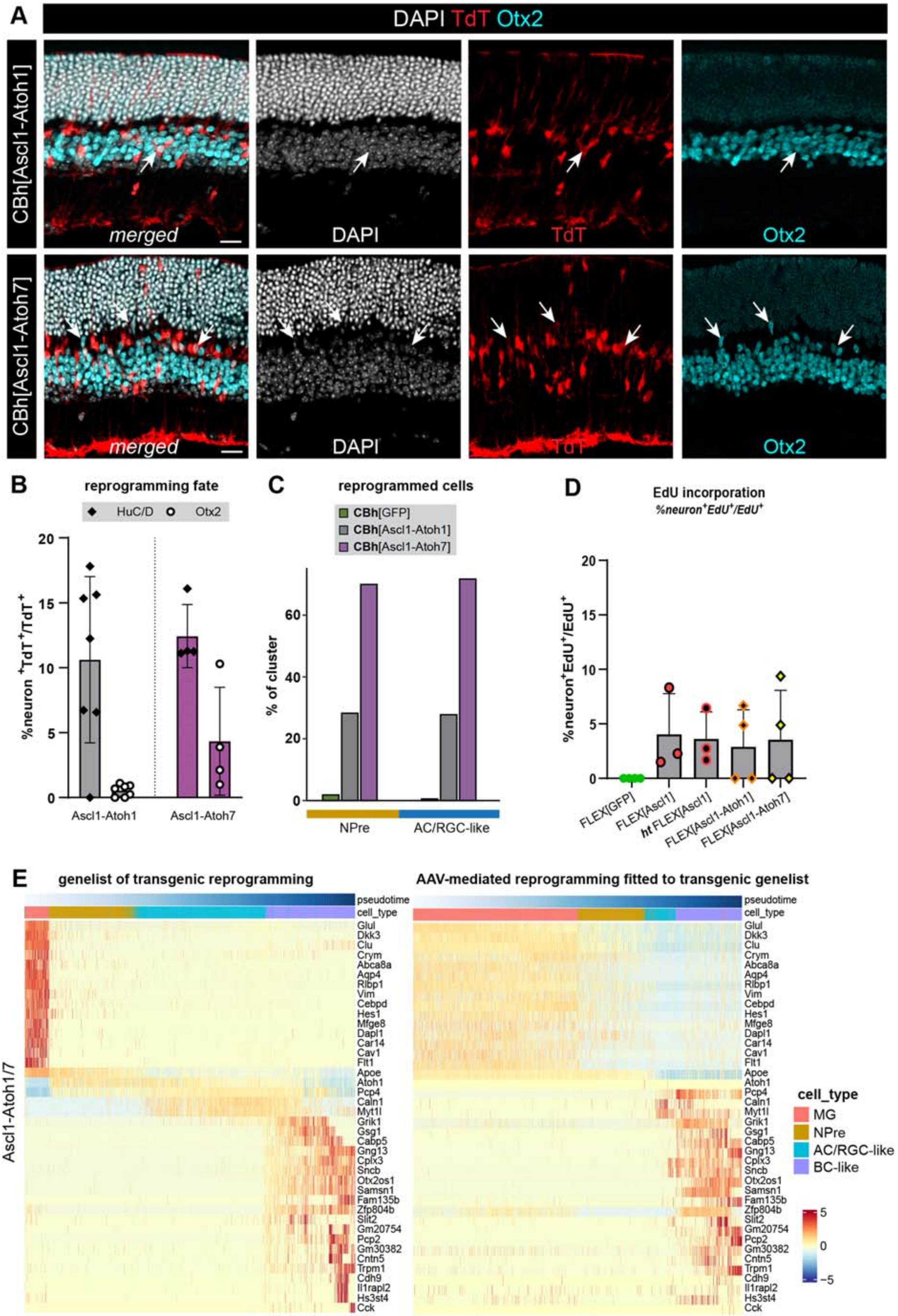
A
DAPI TdT Otx2
CBh[Ascl1-Atoh1]
CBh[Ascl1-Atoh7]
merged
DAPI
TdT
Otx2
B
reprogramming fate
HuC/D
Otx2
%neuron+TdT+/TdT+
Ascl1-Atoh1
Ascl1-Atoh7
C
reprogrammed cells
CBh[GFP]
CBh[Ascl1-Atoh1]
CBh[Ascl1-Atoh7]
% of cluster
NPre
AC/RGC-like
D
EdU incorporation
%neuron+EdU+/EdU+
FLEX[GFP]
FLEX[Ascl1]
ht FLEX[Ascl1]
FLEX[Ascl1-Atoh1]
FLEX[Ascl1-Atoh7]
E
genelist of transgenic reprogramming
AAV-mediated reprogramming fitted to transgenic genelist
pseudotime
cell_type
Ascl1-Atoh1/7
Glul Dkk3 Clu Crym Abca8a Aqp4 Rlbp1 Vim Cebpd Hes1 Mfge8 Dapl1 Car14 Cav1 Flt1 Apoe Atoh1 Pcp4 Caln1 Myt1l Grik1 Gsg1 Cabp5 Gng13 Cplx3 Sncb Otx2os1 Samsn1 Fam135b Zfp804b Slit2 Gm20754 Pcp2 Gm30382 Cntn5 Trpm1 Cdh9 Il1rapl2 Hs3st4 Cck
cell_type
MG
NPre
AC/RGC-like
BC-like

 © The Author(s)

Figure EV5. AAV-borne Ascl1-Atoh1/7 expression induces neurogenesis that phenocopies transgenics.

(**A**, **A′**) Fluorescence images of reprogrammed cells (white arrows) per condition showing merged and single channels of DAPI in white, TdT in red and Otx2 in cyan, (**B**) bar plot of MG reprogramming to distinct neuronal fates (HuC/D or Otx2) after AAV-mediated Ascl1-Atoh1 or Ascl1-Atoh7 expression counted as a percentage ratio of neuron^{+}TdT^{+} over all TdT^{+} cells with each dot being a biological replicate, (**C**) bar plot of the percentrage that each vector treatment contributed to the formation of reprogrammed clusters NPre and MG-derived AC/RGC-like neurons where each column represents a color-coded sample. (**D**) Bar plot of Edu incorporation counted as a ratio of neuron^{+}EdU^{+} over all EdU^{+} cells with each dot being a biological replicate, (**E**) heatmap of top 40 differentially expressed genes across pseudotime trajectory from glial cell fate to neuronal cell fate based on scRNA-seq data from transgenic experiments, followed by heatmap of gene expression in scRNA-seq data from AAV experiments that follows the genelist generated from consolidated transgenic data for Ascl1-Atoh1/7 reprogramming; error bars for bar plots:mean plus standard deviation, statistical significance based on ordinary one-way ANOVA with Tukey's multiple comparisons test (significance $P < 0.05$, * = 0.05, ** = 0.01, *** = 0.001, **** = 0.0001, no significance detected); scale bar for (**A**–**A′**): 20 µm.

© The Author(s)
